# All‐cause and cause‐specific mortality in individuals with an alcohol‐related emergency or hospital inpatient presentation: A retrospective data linkage cohort study

**DOI:** 10.1111/add.16218

**Published:** 2023-05-16

**Authors:** Janni Leung, Vivian Chiu, Nicola Man, Wing See Yuen, Timothy Dobbins, Adrian Dunlop, Natasa Gisev, Wayne Hall, Sarah Larney, Sallie‐Anne Pearson, Louisa Degenhardt, Amy Peacock

**Affiliations:** ^1^ National Drug and Alcohol Research Centre, UNSW Sydney Sydney NSW Australia; ^2^ Faculty of Health and Behavioural Sciences University of Queensland Brisbane QLD Australia; ^3^ School of Population Health UNSW Sydney Sydney NSW Australia; ^4^ Hunter New England Health, New South Wales Government Newcastle NSW Australia; ^5^ Department of Family Medicine and Emergency Medicine University of Montréal Montréal QC Canada; ^6^ Centre de recherche du Centre hospitalier de l'Université de Montréal Montréal QC Canada; ^7^ Medicines Policy Research Unit, Centre for Big Data Research in Health UNSW Sydney Sydney NSW Australia

**Keywords:** Alcohol, emergency department, hospitalization, injury, intoxication, linkage, liver disease, mortality

## Abstract

**Background and Aims:**

Alcohol consumption is a leading risk factor for premature mortality globally, but there are limited studies of broader cohorts of people presenting with alcohol‐related problems outside of alcohol treatment services. We used linked health administrative data to estimate all‐cause and cause‐specific mortality among individuals who had an alcohol‐related hospital inpatient or emergency department presentation.

**Design:**

Observational study using data from the Data linkage Alcohol Cohort Study (DACS), a state‐wide retrospective cohort of individuals with an alcohol‐related hospital inpatient or emergency department presentation.

**Setting:**

Hospital inpatient or emergency department presentation in New South Wales, Australia, between 2005 and 2014.

**Participants:**

Participants comprised 188 770 individuals aged 12 and above, 66% males, median age 39 years at index presentation.

**Measurements:**

All‐cause mortality was estimated up to 2015 and cause‐specific mortality (by those attributable to alcohol and by specific cause of death groups) up to 2013 due to data availability. Age‐specific and age–sex‐specific crude mortality rates (CMRs) were estimated, and standardized mortality ratios (SMRs) were calculated using sex and age‐specific deaths rates from the NSW population.

**Findings:**

There were 188 770 individuals in the cohort (1 079 249 person‐years of observation); 27 855 deaths were recorded (14.8% of the cohort), with a CMR of 25.8 [95% confidence interval (CI) = 25.5, 26.1] per 1000 person‐years and SMR of 6.2 (95% CI = 5.4, 7.2). Mortality in the cohort was consistently higher than the general population in all adult age groups and in both sexes. The greatest excess mortality was from mental and behavioural disorders due to alcohol use (SMR = 46.7, 95% CI = 41.4, 52.7), liver cirrhosis (SMR = 39.0, 95% CI = 35.5, 42.9), viral hepatitis (SMR = 29.4, 95% CI = 24.6, 35.2), pancreatic diseases (SMR = 23.8, 95% CI = 17.9, 31.5) and liver cancer (SMR = 18.3, 95% CI = 14.8, 22.5). There were distinct differences between the sexes in causes of excess mortality (all causes fully attributable to alcohol female versus male risk ratio = 2.5 (95% CI = 2.0, 3.1).

**Conclusions:**

In New South Wales, Australia, people who came in contact with an emergency department or hospital for an alcohol‐related presentation between 2005 and 2014 were at higher risk of mortality than the general New South Wales population during the same period.

## INTRODUCTION

Alcohol is one of the most commonly used psychoactive substances globally. In 2016, 18.2% of people aged ≥ 15 years engaged in heavy episodic drinking (≥ 60 g alcohol in a drinking session) in the past month [1]. Globally, alcohol was ranked the seventh leading risk factor for disease burden in 2016 [2]. This pattern of consumption and harm associated with alcohol is reflected in Australia. One in four (25%) adult Australians drink alcohol at risky levels (> 40 g alcohol on a single occasion) at least monthly [3], and approximately 1.8% of hospitalizations and one in 10 emergency department presentations in Australia are estimated to be alcohol‐related [4, 5].

Alcohol consumption is one of the leading risk factors for mortality [6]. People with an alcohol use disorder have a threefold higher risk of all‐cause mortality compared to the general population [7, 8]. There have been studies on mortality risk associated with alcohol use in the general population [9] and in those receiving treatment for alcohol use disorder [7, 8, 10, 11, 12]. For example, in those seeking treatment for alcohol use disorder, there is a 10‐fold increase in the risk of death from alcohol‐related liver cirrhosis and mental disorders, and a high risk of death from unintentional injuries, suicide, cancers and cardiovascular diseases [12]. However, less is known about mortality among individuals who may be experiencing problems related to their alcohol use who may not necessarily have engaged in formal treatment for an alcohol use disorder. This gap in knowledge is significant, given that only one‐in five Australians with an alcohol use disorder receive treatment [13], with similar findings internationally [14].

Advances in data linkage of routinely collected administrative health‐care data sets with mortality datasets can enable such study, facilitating powerful population‐level analyses of mortality risk with fewer resource implications than cohorts established through primary data collection [15]. Despite this, there have been few studies using such linked data to study mortality risk among people with alcohol‐related problems. Those that have been conducted typically focus on all‐cause or specific causes of death (e.g. acute injury mortality [16, 17, 18]), or specific subpopulations of people presenting to health services with an alcohol‐related diagnosis (e.g. people with a non‐fatal injury with a comorbid alcohol use disorder diagnosis [19]). The exception is a data linkage study of a Danish population‐based cohort of all adults with a first‐time hospital contact for an alcohol‐related diagnosis in 1998–2002 followed for mortality until 2012 [20]. This cohort had four times the rate of all‐cause mortality and 10–34 times the rate of alcohol‐related mortality compared to the general population. This study demonstrates feasibility of using linked administrative data to estimate mortality risk in the broader population of people experiencing alcohol‐related problems, highlighting capacity for similar study in other countries where alcohol is a public health priority, and scope to draw on other health‐care data‐sets (e.g. from emergency departments) where people with alcohol‐related problems are heavily represented [4].

As such, the aims of the current study were to estimate the crude rates of all‐cause and cause‐specific mortality and excess mortality (including by age and sex where possible) in individuals who presented to hospital inpatient and/or emergency departments in New South Wales (NSW), Australia, with an alcohol‐related diagnosis between 2005 and 2014. Given the varying mortality risk across age and sex, we estimated age–sex standardized mortality ratios (SMRs) to study excess mortality in the cohort in comparison with the general Australian population.

## METHOD

### Study design

This is a retrospective cohort study of individuals identified between 2005 and 2014 and followed‐up to 2015 to examine all‐cause mortality, and individuals identified between 2005 and 2012 and followed‐up to 2013 to examine cause‐specific mortality. Data are from the Data linkage Alcohol Cohort Study (DACS), a state‐wide retrospective cohort study established through data linkage of persons in NSW, the most populous Australian state, with an alcohol‐related hospital inpatient or emergency department presentation between 1 January 2005 and 31 December 2014. Details on cohort formation have been published elsewhere [21]. Access was approved by data custodians and linkage was undertaken by the Centre for Health Record Linkage (CHeReL) using Choicemaker software [22]. Ethics approval was provided by the NSW Population and Health Services Research Ethics Committee [21].

### Cohort selection and linkage

Individuals entered the cohort at the time of their first record (‘index presentation’) in the NSW Admitted Patient Data Collection (APDC) or Emergency Department Data Collection (EDDC) with an alcohol‐related diagnosis between 1 January 2005 and 31 December 2014 (see Supporting information, Appendix S1). The index presentation is the first presentation within the given time‐frame and does not necessarily constitute the person's first ever alcohol‐related presentation.

The NSW APDC comprised records of hospital inpatient separations in all public and private hospitals, public multi‐purpose services and day procedure centres in NSW. A separation refers to a completed episode of care for an admitted patient, ending with discharge, death, transfer, leave against medical advice or a portion of a hospital stay beginning or ending in a change to another type of care. A principal diagnosis and up to 50 additional diagnoses are coded according to the International Classification of Diseases, 10th revision, Australian modification (ICD‐10‐AM). All diagnosis fields were analysed.

The NSW EDDC comprises records of emergency department presentations in major metropolitan and non‐metropolitan public hospitals in NSW. The number of emergency departments contributing has increased over time, noting that larger facilities take part, and so data are assumed to capture a substantial proportion of the NSW population [23]. Only a principal diagnosis field is coded in the EDDC. This field was coded according to the International Classification of Diseases and Health Related Problems, 9th revision, clinical modification (ICD‐9‐CM), ICD‐10‐AM or the Systematized Nomenclature of Medicine—clinical terms Australian modification (SNOMED‐CT‐AU).

Alcohol‐related diagnoses used for cohort identification are in Supporting information, Appendix S1. We excluded individuals identified solely on prenatal alcohol exposure or a diagnosis of alcohol in the blood without another alcohol‐related diagnosis (Supporting information, Appendix S1). Individuals were also excluded if they had inconsistent information across data sets (e.g. date of birth/death), were non‐NSW residents or outside the age range 12–100 years at index presentation (see Supporting information, Appendix S2).

### Mortality outcomes

All‐cause mortality data on registered deaths (e.g. age at death, date of death) in NSW were extracted from the NSW Registry of Births, Deaths and Marriages (RBDM) from cohort commencement (i.e. 1 January 2005) to 31 December 2015. Cause‐specific mortality data were obtained for these deaths from the Cause of Death Unit Record File (COD URF). Due to data processing lags, cause‐specific mortality data were only available 1 January 2005 to 31 December 2013. The underlying and all contributory causes of death (up to 20 diagnoses) coded according to ICD‐10 from the COD URF were analysed (Supporting information, Appendix S5).

Mortality data were analysed as fully and/or partly attributable to alcohol (see Supporting information, Appendix S5 for codes); by the corresponding ICD‐10 chapters (i.e. clustered based on body system and/or condition); and by subcategories derived from ICD‐10 codes for mortality fully and/or partly attributable to alcohol (see Supporting information, Appendix S6 for codes). ICD‐10 codes for mortality fully attributable to alcohol comprised alcohol‐induced pseudo‐Cushing's syndrome, Wernicke encephalopathy, mental and behavioural disorders due to alcohol use, degeneration of nervous system due to alcohol, alcoholic polyneuropathy, alcoholic myopathy, alcoholic cardiomyopathy, alcoholic gastritis, alcoholic liver disease, alcohol‐induced pancreatitis and alcohol poisoning [24]. ICD‐10 codes for mortality partly attributable to alcohol were identified from the Australian National Alcohol Indicators Project and existing literature [20, 24, 25, 26].

### General population mortality data

The COD URF data set for all deaths registered in NSW from 1 January 2005 to 31 December 2015 by age (range = 12–100) and sex were obtained from the Australian Bureau of Statistics (2005) and the Australian Coordinating Registry COD URF (2006–15).

### Data analysis

We allowed for 1 year of follow‐up to capture out‐of‐hospital mortality (i.e. mortality that may not be directly related to the index presentation). To allow at least 1 year of follow‐up period from cohort entry to death or censoring the two mortality analyses covered a different study period, as previously detailed. Person‐years of follow‐up were calculated from the index date of admission (cohort entry) until the date of death or censoring (all‐cause mortality = 31 December 2015; cause‐specific mortality = 31 December 2013).

All‐cause crude mortality rates (CMRs; per 1000 population) and indirect SMRs were calculated by age and sex using the observed and expected deaths to produce age‐specific and age–sex‐specific estimates. The expected deaths were calculated by multiplying the person‐years accumulated in the study period by the age–sex‐specific mortality rates (in the 12–15‐ and 16–19‐year age groups, then in 5‐year age groups from 15–19 to 85–89 years and 90+ years) of the standard population [27]. A negative binomial model was fitted for number of observed deaths (in each sex and age group combination) as an outcome and number of expected deaths as exposure to estimate SMR and relative risk (RR) of sex (i.e. females compared with males). A Poisson model was fitted when there was no evidence of overdispersion (i.e. dispersion parameter was not significantly different from 0; see Supporting information, Appendix S7). The estimated SMRs and RRs from both the Poisson and negative binomial models are presented in Supporting information, Appendix S7. SMRs were computed as predicted values from the models. CMRs were calculated as the number of deaths in the cohort divided by person‐years of observation. A Poisson model was used to estimate CMRs and RRs as the negative binomial distribution could not be used when there is only a single data point for computation of a crude rate. Estimates based on numbers < 10 were suppressed in line with the data confidentiality protocol.

Similarly, cause‐specific CMR, SMR and RR were estimated by sex, but not by age, due to small cell sizes after splitting by causes of death for some of the diagnosis groups. The regression modelling was performed in Stata version 16.0 (StataCorp). All other analyses were conducted in SAS version 9.4 [28]. Findings are reported according to the REporting of studies Conducted using Observational Routinely‐collected Data (RECORD) statement [29] (Appendix Supporting information, S9). Statistical significance is determined at *P* < 0.05. Analyses were not pre‐registered; results should be considered exploratory.

## RESULTS

### Sample inclusion

**FIGURE 1:**
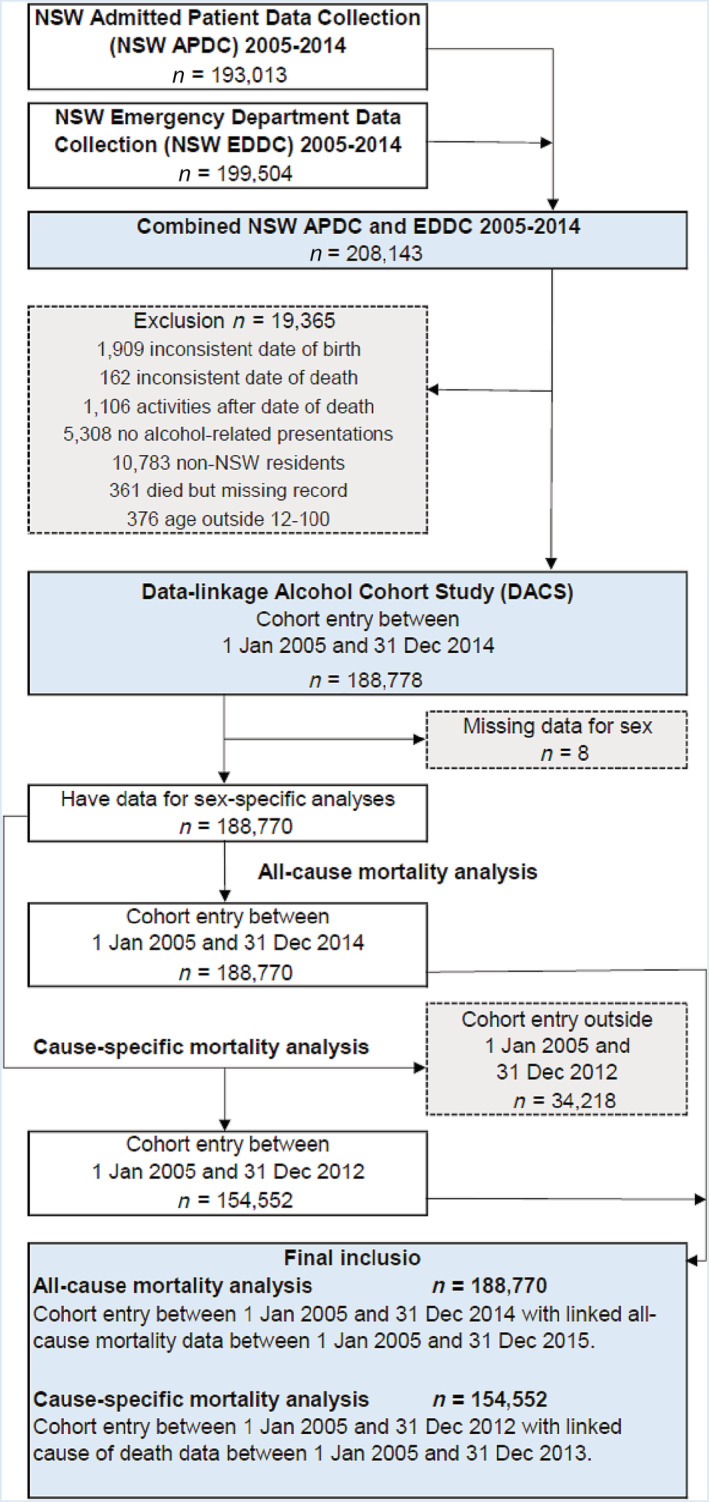
Flow‐chart of participant inclusion.

From the 208 143 individuals identified, 19 365 were excluded for eligibility reasons, leaving 188 778. A further eight with missing data on sex were excluded, therefore the all‐cause mortality analysis included 188 770 individuals who entered the cohort between 1 January 2005 and 31 December 2014, with all‐cause mortality data available to 31 December 2015. The cause‐specific analysis excluded a further 34 218 individuals who did not have at least 1 year of follow‐up data, resulting in the inclusion of 154 552 individuals who entered the cohort between 1 January 2005 and 31 December 2012, with cause‐specific mortality data available to 31 December 2013 (Figure 1).

### Cohort characteristics

Males comprised 66% of the cohort and the median age was 39 years [interquartile range (IQR) = 25, 55] at index presentation (Supporting information, Appendix S3). The most common alcohol‐related diagnoses at index presentation were alcohol intoxication (62.8%), alcohol dependence (23.8%), alcohol withdrawal (8.0%), alcoholic liver disease (5.5%) and toxic effect of alcohol (5.0%; Supporting information, Appendix S4).

### All‐cause mortality

Among the 188 770 individuals in the cohort, there were 27 855 deaths (14.8% of cohort; mean age at death = 65, SD = 16, median = 67, IQR = 55, 78) from 1 079 249 total person‐years of observation. Follow‐up time ranged from 0 to 11 years, with a mean of 5.7 years (SD = 3.1).

Table 1 presents the overall, age‐specific, sex‐specific and age–sex‐specific CMRs and RR of mortality of sex for all deaths in the cohort. The overall CMR was 25.8 per 1000 population (95% CI = 25.5, 26.1). Males (CMR = 30.9, 95% CI = 30.5, 31.3) had a higher CMR than females (CMR = 16.4, 95% CI = 16.0, 16.8; RR = 0.5, 95% CI = 0.5, 0.5; Table 1).

**TABLE 1:** All‐cause mortality: crude (overall), and sex‐, age‐ and age–sex‐specific mortality rate (CMR; per 1000 population) and relative risk (RR) of sex for individuals with an alcohol‐related hospital inpatient or emergency department presentation from 2005 to 2015 (*n* = 188 770).

Age at death	CMR (95% CI)			RR (95% CI)
	Total	Males	Females	Sex (ref: males)
All ages (years)	25.8 (25.5, 26.1)	30.9 (30.5, 31.3)	16.4 (16.0, 16.8)	**0.5 (0.5, 0.5)**
12–24	0.9 (0.8, 1.0)	1.2 (1.0, 1.3)	0.6 (0.5, 0.7)	**0.5 (0.4, 0.6)**
25–34	4.0 (3.7, 4.3)	4.6 (4.3, 5.0)	2.8 (2.4, 3.2)	**0.6 (0.5, 0.7)**
35–44	9.1 (8.7, 9.6)	10.1 (9.6, 10.7)	7.2 (6.6, 7.9)	**0.7 (0.6, 0.8)**
45–54	24.2 (23.5, 25.0)	27.3 (26.3, 28.3)	18.0 (16.9, 19.2)	**0.7 (0.6, 0.7)**
55–64	50.7 (49.4, 52.0)	55.0 (53.4, 56.6)	38.5 (36.3, 40.9)	**0.7 (0.7, 0.7)**
65–74	99.8 (97.3, 102.3)	106.3 (103.5, 109.3)	78.5 (74.1, 83.1)	**0.7 (0.7, 0.8)**
75–84	198.8 (193.9, 203.8)	224.2 (218.0, 230.5)	137.4 (129.9, 145.3)	**0.6 (0.6, 0.7)**
85+	493.2 (475.2, 511.7)	583.6 (558.1, 610.0)	367.0 (343.2, 392.0)	**0.6 (0.6, 0.7)**

Relative risks significant at *P* < 0.05 are highlighted in bold type. Observed and expected number of deaths are available in Supporting information, Appendix S8. CI = confidence interval.

Table 2 presents the excess mortality (SMRs), overall and by age and/or sex, and RR of excess mortality by sex. Mortality was consistently higher than the general population in all adult age groups and in both sexes (overall SMR = 6.2, 95% CI = 5.4, 7.2; Table 2). Overall SMRs were similar between the sexes (RR = 1.0, 95% CI = 0.7, 1.4). However, females had significantly higher SMRs in the 25–3, 35–4 and 55–64‐year age groups and significantly lower SMR in the 85+ age group, compared with males in the corresponding age groups (Table 2).

**TABLE 2:** All‐cause excess mortality: standardized mortality ratios (SMRs) and relative risk (RR) of sex for individuals with an alcohol‐related hospital inpatient or emergency department presentation from 2005 to 2015 (*n* = 188 770).

Age at death	SMR (95% CI)			RR (95% CI)
	Total	Males	Females	Sex (ref: males)
All ages (years)	6.2 (5.4, 7.2)	6.2 (5.2, 7.4)	6.1 (4.6, 8.2)	1.0 (0.7, 1.4)
12–24	2.5 (1.8, 3.5)	2.5 (1.7, 3.6)	2.8 (1.4, 5.8)	1.1 (0.5, 2.6)
25–34	6.7 (5.6, 8.0)	6.4 (5.3, 7.7)	9.1 (7.4, 11.2)	**1.4 (1.1, 1.9)**
35–44	8.4 (7.4, 9.6)	8.0 (7.0, 9.2)	10.5 (10.1, 10.8)	**1.3 (1.1, 1.5)**
45–54	10.0 (9.1, 10.9)	9.7 (8.8, 10.8)	10.9 (9.8, 12.3)	1.1 (1.0, 1.3)
55–64	8.7 (7.8, 9.8)	8.5 (7.5, 9.5)	10.2 (9.9, 10.6)	**1.2 (1.1, 1.4)**
65–74	6.7 (6.0, 7.5)	6.5 (5.8, 7.3)	7.9 (6.7, 9.3)	1.2 (1.0, 1.5)
75–84	4.6 (4.3, 4.9)	4.7 (4.4, 5.0)	4.2 (3.4, 5.2)	0.9 (0.7, 1.1)
85+	4.0 (3.5, 4.6)	4.3 (3.9, 4.8)	3.3 (2.8, 4.0)	**0.8 (0.6, 0.9)**

The SMRs were indirectly standardized by comparing to age–sex‐specific deaths of the general NSW population. The estimated SMRs and RRs were computed in negative binomial models with number of observed deaths as an outcome and number of expected deaths as the exposure variable. RRs significant at *P* < 0.05 are highlighted in bold type. CI = confidence interval.

### Cause‐specific mortality

Of the 154 552 individuals with cause‐specific death data, there were 20 529 deaths throughout 626 770 person‐years of observation. Follow‐up time ranged from 0 to 11 years, with a mean of 4.1 years (SD = 2.4). Table 3 presents the overall and sex‐specific CMRs and RR of mortality of sex for specific causes of deaths in the cohort. CMRs by causes either fully or partly attributable to alcohol were 32.4 (95% CI = 31.8, 32.9) in males and 17.4 (95% CI = 16.8, 18.0) in females. In general, females had lower RR than males for most of the causes.

**TABLE 3:** Cause‐specific mortality: crude (overall) and sex‐specific mortality rate (CMR; per 1000 population) and relative risk (RR) of sex for the alcohol cohort from 2005 to 2013.

Cause of death	CMR (95% CI)			RR (95% CI)
	Total (*N* = 154 552) 626 770 person‐years	Males (*n* = 102 620) 412 152 person‐years	Females (*n* = 51 932) 214 618 person‐years	Sex (ref: males)
Attribution to alcohol^a^				
All causes fully attributable to alcohol	8.3 (8.1, 8.5)	10.0 (9.7, 10.3)	5.1 (4.8, 5.4)	**0.5 (0.5, 0.5)**
All causes partly attributable to alcohol	25.3 (24.9, 25.7)	30.2 (29.6, 30.7)	15.9 (15.4, 16.4)	**0.5 (0.5, 0.5)**
Either fully or partly attributable to alcohol	27.3 (26.8, 27.7)	32.4 (31.8, 32.9)	17.4 (16.8, 18.0)	**0.5 (0.5, 0.6)**
By specific causes of death				
(1) Certain infectious and parasitic diseases (A00–B99)	4.7 (4.5, 4.8)	5.6 (5.4, 5.8)	2.8 (2.6, 3.1)	**0.5 (0.5, 0.5)**
Tuberculosis	<0.1	<0.1	–	–
Viral hepatitis	0.9 (0.8, 0.9)	1.1 (1.0, 1.2)	0.5 (0.4, 0.6)	**0.4 (0.3, 0.5)**
(2) Neoplasms (C00–D48)	8.8 (8.6, 9.1)	11.1 (10.8, 11.5)	4.4 (4.1, 4.7)	**0.4 (0.4, 0.4)**
Breast cancer	–	–	0.7 (0.5, 0.8)	–
Colon and rectal cancer	0.6 (0.6, 0.7)	0.8 (0.8, 0.9)	0.3 (0.2, 0.4)	**0.3 (0.3, 0.4)**
Oesophageal cancer	0.3 (0.3, 0.4)	0.4 (0.4, 0.5)	0.2 (0.1, 0.2)	**0.4 (0.3, 0.6)**
Cancer of lip and oral cavity and pharynx	0.3 (0.3, 0.3)	0.4 (0.3, 0.5)	0.1 (0.1, 0.2)	**0.3 (0.2, 0.5)**
Liver cancer	1.2 (1.1, 1.3)	1.7 (1.6, 1.8)	0.3 (0.2, 0.3)	**0.2 (0.1, 0.2)**
(3) Blood and blood‐forming organs (D50–D89)	1.0 (0.9, 1.1)	1.1 (1.0, 1.2)	0.8 (0.6, 0.9)	**0.7 (0.6, 0.8)**
(4) Endocrine, nutritional and metabolic (E00–E90)	4.6 (4.4, 4.7)	5.6 (5.4, 5.9)	2.5 (2.3, 2.7)	**0.4 (0.4, 0.5)**
Diabetes mellitus	2.9 (2.7, 3.0)	3.7 (3.5, 3.9)	1.3 (1.1, 1.4)	**0.3 (0.3, 0.4)**
(5) Mental and behavioural disorders (F00–F99)	8.2 (8.0, 8.4)	9.5 (9.2, 9.8)	5.8 (5.5, 6.1)	**0.6 (0.6, 0.7)**
Dementia	2.3 (2.2, 2.4)	2.5 (2.3, 2.6)	1.9 (1.7, 2.1)	**0.8 (0.7, 0.8)**
Alcohol use disorders	4.4 (4.2, 4.5)	5.3 (5.1, 5.5)	2.6 (2.4, 2.8)	**0.5 (0.4, 0.5)**
Other substance use disorders	1.6 (1.5, 1.7)	1.8 (1.7, 1.9)	1.2 (1.0, 1.3)	**0.6 (0.6, 0.7)**
(6) Disease of nervous system (G00–G99)	2.2 (2.1, 2.3)	2.6 (2.4, 2.7)	1.4 (1.2, 1.5)	**0.5 (0.5, 0.6)**
Alzheimer's disease	0.3 (0.2, 0.3)	0.3 (0.2, 0.3)	0.3 (0.2, 0.4)	1.1 (0.8, 1.4)
(7) Eye and adnexa (H00–H59)	0.1 (0.0, 0.1)	0.1 (0.0, 0.1)	0.1 (0.0, 0.1)	1.1 (0.6, 2.1)
(9) Diseases of circulatory system (I00–I99)	15.8 (15.4, 16.1)	19.1 (18.7, 19.6)	9.2 (8.8, 9.7)	**0.5 (0.5, 0.5)**
Ischaemic heart diseases	6.2 (6.0, 6.4)	7.8 (7.5, 8.1)	3.2 (2.9, 3.4)	**0.4 (0.4, 0.4)**
Stroke	3.4 (3.3, 3.6)	4.0 (3.8, 4.2)	2.2 (2.0, 2.4)	**0.6 (0.5, 0.6)**
Hypertensive heart disease	0.2 (0.2, 0.2)	0.2 (0.2, 0.3)	0.1 (0.1, 0.2)	**0.5 (0.3, 0.7)**
Cardiomyopathy and myocarditis	1.0 (0.9, 1.1)	1.4 (1.2, 1.5)	0.4 (0.3, 0.4)	**0.3 (0.2, 0.3)**
Atrial fibrillation and flutter	1.7 (1.6, 1.8)	2.0 (1.9, 2.2)	1.0 (0.9, 1.2)	**0.5 (0.4, 0.6)**
Heart failure	2.9 (2.8, 3.1)	3.5 (3.3, 3.7)	1.9 (1.7, 2.0)	**0.5 (0.5, 0.6)**
(10) Diseases of respiratory system (J00–J99)	10.3 (10.1, 10.6)	12.2 (11.8, 12.5)	6.8 (6.5, 7.2)	**0.6 (0.5, 0.6)**
Chronic obstructive pulmonary disease	4.5 (4.3, 4.6)	5.2 (5.0, 5.4)	3.1 (2.9, 3.4)	**0.6 (0.6, 0.7)**
Asthma	0.2 (0.2, 0.3)	0.2 (0.2, 0.3)	0.2 (0.2, 0.3)	1.0 (0.7, 1.4)
(11) Diseases of digestive system (K00–K93)	10.2 (9.9, 10.4)	12.2 (11.9, 12.6)	6.2 (5.8, 6.5)	**0.5 (0.5, 0.5)**
Cirrhosis and other chronic liver diseases	8.4 (8.2, 8.6)	10.2 (9.9, 10.5)	5.0 (4.7, 5.3)	**0.5 (0.5, 0.5)**
Oesophagus, stomach and duodenum	0.8 (0.8, 0.9)	1.1 (1.0, 1.2)	0.5 (0.4, 0.6)	**0.4 (0.3, 0.5)**
Pancreatic diseases	0.5 (0.4, 0.5)	0.5 (0.5, 0.6)	0.3 (0.3, 0.4)	**0.6 (0.5, 0.8)**
(12) Skin and subcutaneous tissue (L00–L99)	0.5 (0.4, 0.5)	0.5 (0.5, 0.6)	0.3 (0.3, 0.4)	**0.7 (0.5, 0.9)**
(13) Musculoskeletal system (M00–M99)	1.0 (0.9, 1.1)	1.0 (0.9, 1.1)	1.0 (0.8, 1.1)	0.9 (0.8, 1.1)
(14) Diseases of genitourinary system (N00–N99)	3.7 (3.6, 3.9)	4.5 (4.3, 4.7)	2.3 (2.1, 2.5)	**0.5 (0.5, 0.6)**
Acute kidney failure and chronic kidney disease	3.3 (3.1, 3.4)	4.0 (3.8, 4.2)	1.9 (1.7, 2.1)	**0.5 (0.4, 0.5)**
(19) Injury and poisoning (S00–T98)	4.8 (4.6, 4.9)	5.4 (5.2, 5.6)	3.5 (3.3, 3.8)	**0.7 (0.6, 0.7)**
Alcohol poisoning	0.6 (0.6, 0.7)	0.7 (0.6, 0.8)	0.6 (0.5, 0.7)	0.8 (0.7, 1.0)
(20) External causes (V01–Y98)	5.1 (4.9, 5.3)	5.8 (5.5, 6.0)	3.7 (3.5, 4.0)	**0.6 (0.6, 0.7)**
Suicide	1.2 (1.1, 1.3)	1.3 (1.2, 1.5)	0.9 (0.8, 1.1)	**0.7 (0.6, 0.8)**
Transport accidents	0.3 (0.3, 0.4)	0.4 (0.4, 0.5)	0.1 (0.1, 0.2)	**0.3 (0.2, 0.5)**
Interpersonal violence	0.1 (0.1, 0.2)	0.1 (0.1, 0.2)	0.1 (0.1, 0.2)	0.7 (0.5, 1.2)

CI = confidence interval; – = not reported due to protocol of suppression of cell sizes *n* < 10. Relative risks significant at *P* < 0.05 are highlighted in bold type.

^a^ See Supporting information, Appendix S5 for list of diseases counted towards causes attributable to alcohol; fully and partly are not mutually exclusive.

#### Total by causes defined as fully and partly attributable to alcohol

Table 4 presents the excess mortality (SMRs), overall and by sex, and RR of excess mortality of sex, for specific causes of deaths. The cohort had elevated mortality in causes fully attributable to alcohol (SMR = 50.3, 95% CI = 44.6, 56.8; Table 4). Crude mortality for causes fully attributable to alcohol was lower in females than males (CMR = 5.1 in females versus 10.0 in males; RR = 0.5, 95% CI = 0.5, 0.5; Table 3), but females had higher excess mortality from causes of deaths fully attributable to alcohol, compared with males (SMR = 110.2 in females versus 44.7 in males; RR = 2.5, 95% CI = 2.0, 3.1; Table 4).

**TABLE 4:** Cause‐specific excess mortality: standardized mortality ratios (SMRs) and relative risk (RR) of sex for the alcohol cohort from 2005 to 2013.

Cause of death	SMR (95% CI)			RR (95% CI)
	Total (*N* = 154 552) 626 770 person‐years	Males (*n* = 102 620) 412 152 person‐years	Females (*n* = 51 932) 214 618 person‐years	Sex (ref: males)
Attribution to alcohol^a^				
All causes fully attributable to alcohol	50.3 (44.6, 56.8)	44.7 (40.2, 49.7)	110.1 (90.7, 133.6)	**2.5 (2.0, 3.1)**
All causes partly attributable to alcohol	7.5 (6.1, 9.3)	7.6 (6.0, 9.8)	7.1 (4.9, 10.3)	0.9 (0.6, 1.5)
Either fully or partly attributable to alcohol	8.0 (6.4, 10.0)	8.1 (6.2, 10.5)	7.7 (5.2, 11.5)	1.0 (0.6, 1.5)
By specific causes of death				
(1) Certain infectious and parasitic diseases (A00–B99)	11.2 (8.3, 15.1)	11.3 (8.0, 16.0)	10.8 (6.6, 17.7)	1.0 (0.5, 1.7)
Tuberculosis	5.6 (3.6, 8.8)^†^	5.9 (3.7, 9.6)^†^	–	–
Viral hepatitis	29.4 (24.6, 35.2)	29.2 (22.5, 37.8)	49.2 (41.4, 58.4)	**1.7 (1.3, 2.1)**
(2) Neoplasms (C00–D48)	4.8 (4.2, 5.5)	5.0 (4.2, 5.9)	4.1 (3.5, 4.7)	0.8 (0.7, 1.0)
Breast cancer	3.2 (2.4, 4.2)	–	3.2 (2.4, 4.2)	–
Colon and rectal cancer	3.7 (3.3, 4.0)^†^	3.9 (3.5, 4.3)^†^	2.7 (2.1, 3.5)^†^	**0.7 (0.5, 0.9)** ^†^
Oesophageal cancer	5.3 (4.2, 6.7)	5.2 (4.1, 6.7)	8.0 (4.4, 14.5)	1.5 (0.8, 2.9)
Cancer of lip and oral cavity and pharynx	13.2 (11.0, 15.8)	13.1 (10.9, 15.9)	15.4 (9.5, 24.9)	1.2 (0.7, 2.0)
Liver cancer	18.3 (14.8, 22.5)	19.0 (15.3, 23.6)	10.0 (6.5, 15.4)	**0.5 (0.3, 0.9)**
(3) Blood and blood‐forming organs (D50–D89)	7.3 (5.5, 9.8)	7.3 (5.2, 10.1)	7.7 (4.5, 13.2)	1.1 (0.6, 2.0)
(4) Endocrine, nutritional and metabolic (E00–E90)	6.4 (5.1, 8.0)	6.7 (5.2, 8.6)	5.1 (3.5, 7.6)	0.8 (0.5, 1.2)
Diabetes mellitus	5.8 (4.6, 7.4)	6.2 (4.8, 8.1)	4.0 (2.5, 6.2)	0.6 (0.4, 1.1)
(5) Mental and behavioural disorders (F00–F99)	13.0 (9.9, 17.2)	13.4 (9.7, 18.4)	12.0 (6.9, 20.6)	0.9 (0.5, 1.7)
Dementia	6.2 (5.3, 7.3)	6.7 (5.5, 8.1)	5.2 (4.2, 6.4)	0.8 (0.6, 1.0)
Alcohol use disorders	46.7 (41.4, 52.7)	41.9 (37.7, 46.5)	112.0 (92.9, 135.1)	**2.7 (2.2, 3.3)**
Other substance use disorders	13.2 (10.9, 15.9)	12.0 (9.9, 14.5)	23.4 (18.3, 30.1)	**2.0 (1.4, 2.7)**
(6) Disease of nervous system (G00–G99)	5.0 (3.9, 6.5)	5.2 (3.8, 7.1)	4.4 (3.2, 5.9)	0.8 (0.5, 1.3)
Alzheimer's disease	3.3 (2.8, 3.8)^†^	3.4 (2.8, 4.1)^†^	3.1 (2.4, 4.0)^†^	0.9 (0.7, 1.3)^†^
(7) Eye and adnexa (H00–H59)	5.1 (3.8, 6.9)^†^	4.8 (3.3, 7.1)^†^	5.6 (3.4, 9.3)^†^	1.2 (0.6, 2.2)^†^
(9) Diseases of circulatory system (I00–I99)	6.2 (5.1, 7.4)	6.3 (5.1, 7.9)	5.5 (3.9, 7.6)	0.9 (0.6, 1.3)
Ischaemic heart diseases	5.1 (4.3, 6.1)	5.2 (4.3, 6.3)	4.6 (3.2, 6.6)	0.9 (0.6, 1.3)
Stroke	5.8 (4.7, 7.2)	6.2 (4.8, 8.0)	4.5 (3.1, 6.4)	0.7 (0.5, 1.1)
Hypertensive heart disease	5.5 (4.1, 7.4)	5.9 (4.4, 8.0)	3.2 (1.9, 5.4)	**0.5 (0.3, 1.0)**
Cardiomyopathy and myocarditis	11.3 (9.3, 13.6)	11.6 (9.5, 14.1)	7.7 (4.5, 13.0)	0.7 (0.4, 1.2)
Atrial fibrillation and flutter	6.0 (5.2, 6.9)	6.3 (5.3, 7.5)	4.6 (3.6, 5.9)	**0.7 (0.5, 1.0)**
Heart failure	5.7 (4.8, 6.9)	6.1 (4.9, 7.5)	4.5 (3.4, 5.9)	0.7 (0.5, 1.0)
(10) Diseases of respiratory system (J00–J99)	7.4 (6.2, 8.9)	7.3 (5.9, 9.1)	7.8 (5.6, 10.9)	1.1 (0.7, 1.6)
Chronic obstructive pulmonary disease	9.6 (7.7, 12.0)	8.9 (7.0, 11.4)	12.7 (9.5, 17.0)	1.4 (1.0, 2.1)
Asthma	5.6 (4.4, 7.0)	5.8 (4.5, 7.5)	5.0 (3.0, 8.2)	0.9 (0.5, 1.5)
(11) Diseases of digestive system (K00–K93)	21.2 (16.5, 27.2)	21.2 (16.0, 28.2)	21.1 (13.0, 34.2)	1.0 (0.6, 1.7)
Cirrhosis and other chronic liver diseases	39.0 (35.5, 42.9)	36.7 (33.4, 40.5)	56.1 (45.9, 68.5)	**1.5 (1.2, 1.9)**
Oesophagus, stomach and duodenum	11.7 (8.4, 16.4)	12.4 (8.5, 18.0)	8.3 (4.4, 15.7)	0.7 (0.3, 1.4)
Pancreatic diseases	23.8 (17.9, 31.5)	23.7 (17.4, 32.2)	24.4 (12.7, 47.0)	1.0 (0.5, 2.1)
(12) Skin and subcutaneous tissue (L00–L99)	7.8 (5.8, 10.5)	8.2 (5.7, 11.7)	6.3 (4.4, 9.0)	0.8 (0.5, 1.2)
(13) Musculoskeletal system (M00–M99)	5.8 (5.0, 6.8)	6.1 (5.1, 7.4)	4.9 (3.7, 6.4)	0.8 (0.6, 1.0)
(14) Diseases of genitourinary system (N00–N99)	6.3 (5.0, 8.0)	6.5 (4.9, 8.6)	5.6 (3.9, 8.2)	0.9 (0.5, 1.4)
Acute kidney failure and chronic kidney disease	6.5 (5.1, 8.3)	6.7 (5.0, 8.9)	5.6 (3.8, 8.3)	0.8 (0.5, 1.3)
(19) Injury and poisoning (S00–T98)	9.2 (7.9, 10.8)	8.4 (7.2, 9.9)	13.4 (9.2, 19.5)	**1.6 (1.0, 2.4)**
Alcohol poisoning	26.0 (21.1, 32.0)	24.3 (21.7, 27.3)^†^	58.2 (48.7, 69.6)^†^	**2.4 (1.9, 3.0)** ^†^
(20) External causes (V01–Y98)	8.8 (7.5, 10.3)	8.1 (6.9, 9.5)	12.3 (8.5, 17.9)	**1.5 (1.0, 2.3)**
Suicide	9.1 (8.1, 10.3)	8.5 (7.5, 9.6)	18.7 (13.7, 25.5)	**2.2 (1.6, 3.1)**
Transport accidents	3.8 (3.1, 4.8)	3.8 (3.0, 4.8)	3.8 (2.0, 7.1)	1.0 (0.5, 1.9)
Interpersonal violence	7.9 (5.7, 11.1)	7.7 (5.9, 10.0)^†^	13.6 (8.9, 20.6)^†^	**1.8 (1.1, 2.9)** ^†^

The standardized mortality ratios were indirectly standardised by comparing to age–sex‐specific deaths of the general NSW population. The estimated SMRs and RRs were computed in count regression models with number of observed deaths as an outcome and number of expected deaths as the exposure variable. The negative binomial model was used except where there was no evidence of overdispersion as indicated by † in the table in which case a Poisson model was used (see Supporting information, Appendix S7). Relative risks significant at *P* < 0.05 are highlighted in bold. CI = confidence interval; – = not reported due to protocol of suppression of cell sizes *n* < 10.

^a^ See Supporting information, Appendix S5 for list of diseases counted towards causes attributable to alcohol; fully and partly are not mutually exclusive.

#### Cause‐specific mortality

Excess mortality was observed across all ICD‐10 chapters. The greatest excess mortality was due to alcohol use disorders (SMR = 46.7, 95% CI = 41.4, 52.7), followed by diseases of the digestive system: cirrhosis and other chronic liver disease (SMR = 39.0, 95% CI = 35.5, 42.9), pancreatic diseases (SMR = 23.8, 95% CI = 17.9, 31.5), viral hepatitis (SMR = 29.4, 95% CI = 24.6, 35.2) and liver cancer (SMR = 18.3, 95% CI = 14.8, 22.5). Deaths associated with heart diseases also had high excess mortality: cardiomyopathy and myocarditis (SMR = 11.3, 95% CI = 9.3, 13.6), stroke (SMR = 5.8, 95% CI = 4.7, 7.2), heart failure (SMR = 5.7, 95% CI = 4.8, 6.9) and ischaemic heart diseases (SMR = 5.1, 95% CI = 4.3, 6.1). Excess mortality associated with alcohol poisoning (SMR = 26.0, 21.1, 32.0), suicide (SMR = 9.1, 95% CI = 8.1, 10.3) and interpersonal violence (SMR = 7.9, 95% CI = 5.7, 11.1) were also elevated compared to the general population.

Compared to the general population, excess mortality of females was significantly higher than that of males for deaths caused by alcohol use disorders (RR = 2.7, 95% CI = 2.2, 3.3) or other substance use disorders (RR = 2.0, 95% CI = 1.4, 2.7), alcohol poisoning (RR = 2.4, 95% CI = 1.9, 3.0), suicides (RR = 2.2, 95% CI = 1.6, 3.1), interpersonal violence (RR = 1.8, 1.1, 2.9), viral hepatitis (RR = 1.7, 95% CI = 1.3, 2.1) and cirrhosis and other chronic liver disease (RR = 1.5, 95% CI = 1.2, 1.9). Females had significantly lower SMRs than males from deaths caused by liver cancer (RR = 0.5, 95% CI = 0.3, 0.9), hypertensive heart disease (RR = 0.5, 95% CI = 0.3, 1.0), atrial fibrillation and flutter (RR = 0.7, 95% CI = 0.5, 1.0) and colon and rectal cancer (RR = 0.7, 95% CI = 0.5, 0.9).

## DISCUSSION

This study examined mortality risk using population‐level linked data for a cohort of individuals who presented with an alcohol‐related hospital inpatient or emergency department presentation between 2005 and 2014 in NSW, Australia. All‐cause mortality risk was six times higher than the general population overall. The greatest excess mortality risk arose from mental and behavioural disorders due to alcohol use, liver cirrhosis, viral hepatitis, pancreatic diseases and liver cancer. Females had twice the male excess mortality due to alcohol and other substance‐related disorders, alcohol poisoning and suicides. Males had almost double the excess mortality risk for mortality due to liver cancer and hypertensive heart disease. This population experienced disproportionate mortality risk, often from causes which can be wholly or partially attributed to alcohol use; their attendance at acute health‐care services represents a critical opportunity to identify their problem alcohol use and intervene to reduce mortality risks.

This study was novel in its (i) use of multiple acute health‐care service data‐sets linked with mortality data, (ii) study of an Australian cohort and (iii) focus on a broader population of people with alcohol‐related problems than only those engaged in alcohol treatment. Observed mortality risk was higher than the all‐cause mortality risk of four times the general population observed in the study by Askgaard *et al*. [20] of Danish adults with a first‐time hospital contact for an alcohol‐related diagnosis. The latter study concentrated on adults aged 20–80 years (12–100 in the current study) and with follow‐up for more than 10 years (minimum 1 year in the current study). Further, only one Australian study, which was from the 1960s of a small number of patients with liver cirrhosis [30], was included in the aforementioned systematic reviews. Without comparative Australian studies, it is difficult to attribute the difference in mortality risk to country, although high rates of heavy alcohol consumption in Australia relative to other countries [31] lends weight to this as a potential explanation along with sample characteristics and design differences.

Excess mortality was observed across all causes studied but was predominantly driven by diseases associated with chronic heavy alcohol use (e.g. alcohol‐related mental and behavioural disorders, cardiovascular diseases, liver and pancreatic diseases). This accords with previous work [20], and reinforces the opportunity that acute health‐care services have to identify these individuals and intervene to address chronic patterns of alcohol use and accompanying health risks. Excess mortality was also observed for acute injury‐related causes (e.g. suicide, interpersonal violence) arising from alcohol intoxication. This matches findings from a 1‐year follow‐up study of acute injury mortality among people in California who attended an emergency department (ED) and who had an alcohol use disorder (AUD) diagnosis, although SMRs were higher in the current study [16].

While small cell sizes prevented us from studying differences by age group, the aforementioned Californian study found very high risks for suicide and homicide in those aged 10–45 years [16]. This suggests that strategies to reduce mortality risk may need to be tailored to age group. Similarly, the current study showed that women had greater excess acute injury mortality risk, while males had greater excess risk due to cardiovascular causes and certain cancers. This accords with existing literature [12, 16, 20] and reinforces importance of considering demographic differences in mortality risk, with scope in future work to consider other factors (e.g. socio‐economic deprivation [32]).

### Implications

People presenting to emergency department and hospital with an alcohol‐related problem are at increased risk of premature mortality. This point of service contact may represent a unique opportunity to identify and intervene to potentially reduce such risk. Current Australian alcohol treatment guidelines recommend that emergency departments and hospitals should screen to identify risk alcohol use, offer brief interventions in certain circumstances and refer to general practitioner and other services for ongoing monitoring of alcohol use and interventions [33].

Given socio‐demographic differences in excess mortality risk across injury and natural cause in cause‐specific mortality risk, linking people with potential alcohol use disorder with person‐centred, integrated care which addresses alcohol use and other co‐occurring health issues (e.g. alcohol use alongside other mental health problems) may assist in reducing mortality risk [34]. Understanding demographic differences in risk by sex can also help inform the shape of interventions; for example, these findings reinforce the importance of overdose and suicide prevention strategies for this population, particularly for females. In saying this, it is important to acknowledge the significant barriers facing health professionals in emergency departments and hospitals in screening, providing brief intervention and/or referring to other services for further treatment [35].

In‐hospital alcohol consultation liaison services are established with the goal of improving identification of people experiencing alcohol‐related harms and facilitating direct access to specialist services for support and treatment advice [36]. Although resourcing of such systems is a challenge, alcohol consultation liaison services reduce the burden for front‐line medical staff, and provide a clear pathway for ensuring appropriate care within hospital and post‐discharge [36]. Indeed, an economic analysis suggested that alcohol consultation liaison services in NSW, Australia, would save at least $100 000 per hospital per year on average by reducing the average length of hospital stay and decreasing frequency of admission over time [36]. Similar outcomes have been observed for dedicated drug and alcohol brief intervention teams within emergency departments in other Australian jurisdictions [37], reinforcing the value of further study of outcomes from such services. Indeed, overall there is a need for research that evaluates the impact of various strategies deployed within these settings in reducing mortality risk to inform future policy and resource allocation.

### Limitations

There are several limitations to note. Data from the current study are drawn from one Australian jurisdiction (albeit the most populous one) from 2005 to 2015. Findings may not be generalizable to other jurisdictions or countries, nor to other time‐periods. Future work using data over a longer and more recent time‐period would assist in in identifying a person's first ever presentation and changes in clinical risk over time. In addition, future research could examine potential individual patient characteristic factors that may be associated with increased risks of mortality following an alcohol‐related presentation. At presentation, health‐care professionals collect a range of additional information about the patient in their medical records. Future research that makes use of this additional patient‐level information collected to identify potential predictors of negative future outcomes are warranted.

While our population‐level sample was large, under‐ascertainment of cases is probable, given that we could only determine alcohol involvement via diagnostic code and alcohol‐related diagnoses are often not identified nor coded in emergency department and hospitals [38]. This is particularly pertinent for emergency department data, where only one diagnosis field is available, so alcohol‐related mortality risks may have been underestimated. Mortality rates may also be underestimated, as data on deaths that occurred outside NSW were not linked. There may also be a risk of bias from missing records due to people moving and/or dying in another jurisdiction. We attempt to mitigate this bias by computing SMR‐based mortality data for all NSW residents. Finally, although we studied causes partly attributable to mortality, we cannot definitively attribute these to alcohol.

## CONCLUSION

All‐cause mortality among individuals with an alcohol‐related hospital inpatient or emergency department presentation was substantially elevated over that of the general population. Cause‐specific analyses showed that greatest excess mortality was in deaths caused by alcohol‐related mental and behavioural disorders, liver cirrhosis, pancreatic diseases, viral hepatitis and liver cancer. Accurate estimation of excess mortality is important in informing health service delivery and policy planning in Australia. We need to evaluate and strengthen strategies to identify and support people with alcohol‐related problems in hospital and emergency departments to reduce mortality risks.

## AUTHOR CONTRIBUTIONS

**Janni Leung:** Conceptualization (equal); methodology (lead); supervision (lead); writing—original draft (equal). **Vivian Chiu:** Formal analysis (lead); writing—original draft (equal). **Nicola Man:** Methodology (supporting); writing—review and editing (supporting). **Wing See Yuen:** Writing—review and editing (supporting). **Timothy Dobbins:** Writing—review and editing (supporting). **Adrian Dunlop:** Writing—review and editing (supporting). **Natasa Gisev:** Writing—review and editing (supporting). **Wayne Hall:** Writing—review and editing (supporting). **Sarah Larney:** Writing—review and editing (supporting). **Sallie‐Anne Pearson:** Writing—review and editing (supporting). **Louisa Degenhardt:** Writing—review and editing (supporting). **Amy Peacock:** Conceptualization (equal); funding acquisition (lead); resources (lead); writing—review and editing (supporting).

## DECLARATION OF INTERESTS

A.P. has received an untied educational grant from Seqirus and Mundipharma for study of opioid medications. L.D. has received anuntied educational grant from Seqirus, Indivior and Mundipharma for study of opioid medications. In 2020, the Centre for Big Data Research in Health received funding from AbbVie Australia to conduct post‐market surveillance research. These organizations did not have any knowledge of, or involvement in, the current study. The authors declare no conflicts of interest.

## Supporting information


**Appendix S1.** Diagnostic codes for identifying people who had presented to the hospital or emergency department for an alcohol‐related diagnosis.
