# Supplementary material for: All‐cause and cause‐specific mortality in individuals with an alcohol‐related emergency or hospital inpatient presentation: A retrospective data linkage cohort study
Source: Addiction. 2023 May 16;118(9):1751–62. doi: 10.1111/add.16218 (PMC10952273; doi:10.1111/add.16218)
Supplement: Supplementary file 1 — Appendix S1. Diagnostic codes for identifying people who had presented to the hospital or emergency department for an alcohol‐related diagnosis. [file ADD-118-1751-s001.docx]

**Supplementary Appendix - All-cause and cause-specific mortality in individuals with an alcohol-related inpatient or emergency department presentation: a data-linkage cohort study**

Janni Leung, Vivian Chiu, Nicola Man, Wing See Yuen, Timothy Dobbins, Adrian Dunlop, Natasa Gisev, Wayne Hall, Sarah Larney, Sallie-Anne Pearson, Louisa Degenhardt, Amy Peacock (2023)

**Table of contents**

[Appendix 1. Diagnostic codes for identifying people who had presented to the hospital or emergency department for an alcohol-related diagnosis 2](#_Toc124496151)

[1.1. ICD codes 2](#_Toc124496152)

[1.2. SNOMED codes 5](#_Toc124496153)

[Appendix 2. Cohort formation of The Data-linkage Alcohol Cohort Study (DACS). 22](#_Toc124496154)

[NSW Admitted Patient Data Collection (APDC) 22](#_Toc124496155)

[Emergency Department Data Collection (EDDC) 22](#_Toc124496156)

[Cohort inclusion 23](#_Toc124496157)

[Appendix 3. Characteristics of individuals first presented to hospital or emergency department with an alcohol-related problem between 2005-2014, and deaths to up until December 31^st^, 2015 (N=188,770) 25](#_Toc124496158)

[Appendix 4. Alcohol-related problem participants presented with at cohort entry 26](#_Toc124496159)

[Figure A4.1. Treemap of alcohol-related problems (ICD-10 codes) participants presented with at cohort entry 26](#_Toc124496160)

[Table A4.1. Alcohol-related problems participants presented with at cohort entry 27](#_Toc124496161)

[Appendix 5. Causes of deaths attributable to alcohol 28](#_Toc124496162)

[Table A5.1. All causes of deaths fully attributable to alcohol 28](#_Toc124496163)

[Table A5.2. All-causes of deaths partly attributable to alcohol 29](#_Toc124496164)

[References 32](#_Toc124496165)

[Appendix 6. ICD-10 diagnostic codes for the disease sub-categories of cause-specific mortality ratios estimated 31](#_Toc124496166)

[Appendix 7. Count regression models for analysis of excess mortality 33](#_Toc124496167)

[Table A7.1 Overdispersion parameter in the negative binomial models for analysis of excess mortality 33](#_Toc124496168)

[Table A7.2 Total - Estimated overall standardised mortality ratio (SMR) in the Poisson and negative binomial models 35](#_Toc124496169)

[Table A7.3 By sex - Estimated standardised mortality ratio (SMR) and relative risk (RR) of sex in the Poisson and negative binomial models 37](#_Toc124496170)

[Appendix 8. Number of deaths, percent of total and age-sex-standardised mortality rate (per 1,000 population) 40](#_Toc124496171)

[Table A8.1. Number of all-cause deaths for individuals with an alcohol-related hospital inpatient or emergency department presentation from 2005 to 2015 (N= 188,770). 40](#_Toc124496172)

[Table A8.2. Number of cause-specific deaths for the alcohol cohort from 2005 to 2013. 41](#_Toc124496173)

[Appendix 9. RECODE statement checklist 44](#_Toc124496174)

# Appendix 1. Diagnostic codes for identifying people who had presented to the hospital or emergency department for an alcohol-related diagnosis

## 1.1. ICD codes

The codes in Table A1.1 were used to identify people in the Admitted Patients Data Collection and Emergency Department Data Collection (cases). Codes were selected based on identification by NSW Health as reflective of an acute alcohol problem [1] or, where indicated (*) from Turning Point Alcohol and Drug Centre guidance for calculating alcohol and other drug statistics [2]. Where indicated (~), additional codes are included on the basis of discussion between investigators and specialists in the field. ICD-9-CM and ICD-10-AM codes were mapped using the Australian Consortium for Classification Development mapping tables, with backwards mapping from ICD-10-AM to ICD-9-CM [3], and alcohol-specific code mapping developed by Chikritzhs et al. [4].

### References

1. Toson B, Harvey LA, Close JC. New ICD-10 version of the Multipurpose Australian Comorbidity Scoring System outperformed Charlson and Elixhauser comorbidities in an older population. J Clin Epidemiol 2016;79:62-69. doi: 10.1016/j.jclinepi.2016.04.004 [published Online First: 2016/04/23]

2. Preen DB, Holman CD, Spilsbury K, et al. Length of comorbidity lookback period affected regression model performance of administrative health data. J Clin Epidemiol 2006;59(9):940-6. doi: 10.1016/j.jclinepi.2005.12.013 [published Online First: 2006/08/10]

3. NSW. IaPC. Statutory Guidelines on Research: Health Records and Information Privacy Act 2002 (NSW). 2002

4. Chikritzhs TC, P.; Stockwell, T.; Donath, S.; Ngo, H.; Young, D. and Mathews, S. Australian alcohol indicators, 1990-2001: Patterns of alcohol use and related harms for Australian states and territories. : Curtin University of Technology: National Drug Research Institute, 2003.

### Table A1.1. ICD codes and corresponding conditions

| **ICD-10-AM** | **Conditions** | **ICD-9-CM** | **Conditions** |
| --- | --- | --- | --- |
| E24.4 | *Alcohol-induced pseudo-Cushing's syndrome | *No alcohol-specific code available* | |
| E51.2 | ~Wernicke encephalopathy | 291.1 | Alcohol-induced persisting amnestic disorder |
| F10 | Mental and behavioural disorders due to use of alcohol | 291 | Alcohol-induced mental disorders |
|  |  | 303 | Alcohol dependence syndrome |
|  |  | 305.0 | Nondependent alcohol abuse |
| G31.2 | *Degeneration of nervous system due to alcohol | 303 | Alcohol dependence syndrome |
| G62.1 | *Alcoholic polyneuropathy | 357.5 | *Alcoholic polyneuropathy |
| G72.1 | *Alcoholic myopathy | *No alcohol-specific code available* | |
| I42.6 | *Alcoholic cardiomyopathy | 425.5 | *Alcoholic cardiomyopathy |
| K29.2 | *Alcoholic gastritis | 535.3 | *Alcoholic gastritis |
| K70.0 | Alcoholic fatty liver | 571.0 | *Alcoholic fatty liver |
| K70.1 | Alcoholic hepatitis | 571.1 | Acute alcoholic hepatitis |
| K70.2 | Alcoholic fibrosis and sclerosis of liver |  |  |
| K70.3 | Alcoholic cirrhosis of liver | 571.2 | Alcoholic cirrhosis of liver |
| K70.4 | Alcoholic hepatic failure |  |  |
| K70.9 | Alcoholic liver disease, unspecified | 571.3 | Alcoholic liver damage, unspecified |
| K85.2 | *Alcohol-induced acute pancreatitis | *No alcohol-specific code available* | |
| K86.0 | *Alcohol-induced chronic pancreatitis | *No alcohol-specific code available* | |
| O35.4 | *Maternal care for suspected damage to foetus from alcohol | *No alcohol-specific code available* | |
| P04.3 | *Foetus and newborn affected by maternal use of alcohol | 760.71 | Alcohol affecting fetus or newborn via placenta or breast milk |
| Q86.0 | *Foetal alcohol syndrome (dysmorphic) |  |  |
| R78.0 | Finding of alcohol in blood | 790.3 | Excessive blood level of alcohol |
| T51 | Toxic effect of alcohol | 980 | Toxic effect of alcohol |
| X45 | Accidental poisoning by and exposure to alcohol | E860 | Accidental poisoning by alcohol not elsewhere classified |
| X65^^^ | Intentional self-poisoning by and exposure to alcohol | *No alcohol-specific code available* | |
| Y15 | Poisoning by and exposure to alcohol, undetermined intent | E860 | Accidental poisoning by alcohol not elsewhere classified |
|  |  | 980 | Toxic effect of alcohol |
| Y90^^^^ | Evidence of alcohol involvement determined by blood alcohol level | *No alcohol-specific code available* | |
| Y91^^^^^ | Evidence of alcohol involvement determined by level of intoxication | *No alcohol-specific code available* | |
| *Individuals with records of the * diagnostic codes only were excluded due to either prenatal exposure to alcohol or insufficient evidence to indicate that alcohol played a critical role for the episode of care provided.  ^note that no generalised mapping matches were available with SNOMED although lexical matching suggest use of the codes in Table A1.2  ^^note that no generalised mapping matches were available with SNOMED although lexical matching suggest use of the 'Finding of alcohol in blood' code  ^^^note that generalised mapping matches were only available for Y91.1 and Y91.9 with SNOMED although lexical matching suggest use of 'Alcohol intoxication' code | | | |

## 1.2. SNOMED codes

The codes presented in Table A1.2 were used to identify people in the Emergency Department Data Collection (cases). Emergency departments may use either or a combination of ICD-9, ICD-10 or SNOMED. Codes were mapped in consultation with the Clinical Terminology Team at the National E-Health Transition Authority (now Australian Digital Health Agency) to approximate ICD-10-AM codes using lexical and generalised mapping (the latter comprising the International SCT-ICD map) for SCT-AU (v20160430 April 2016). A number of codes were only found in ICD-10 and not ICD-10-CM (F10 K29.20 K29.21 T51 X45 X65 Y90 Y91), and thus these codes were searched with a wildcard for the last character, yielding hits for the following codes (F10.0 F10.1 F10.2 F10.3 F10.4 F10.5 F10.6 F10.7 F10.8 F10.9 K29.2 T51.0 T51.1 T51.2 T51.3 T51.9 X45 X45.99 Y91.1 Y91.9). Lexical mapping (i.e., synonym with a lexical match) was used for those codes where a hit was not identified with the International SCT-ICD map. Note that those variables flagged with a “#” were added following review of NSW Health codes for acute emergency department presentation data; the same applies where flagged with a “^”, with the exception that these terms are now deprecated.

### Table A1.2. SNOMED codes and corresponding conditions mapped to approximate ICD codes

| **ICD-10-AM** | **ICD-9-CM** | **SNOMED-CT-AU** | **CONDITIONS** |
| --- | --- | --- | --- |
| E24.4 | *No alcohol-specific code available* | 237738005 | Pseudo-Cushing's syndrome due to alcohol |
| E51.2 | 291.1 | 21007002 | Wernicke's disease |
| F10 | 291 | 191477001 | Pathological alcohol intoxication |
|  | 303 | 42344001 | Alcohol-induced psychosis |
|  | 305.0 | 25702006 | Alcohol intoxication |
|  |  | 228315001 | Binge drinker |
|  |  | 18653004 | Alcohol intoxication delirium |
|  |  | 21000000 | Idiosyncratic intoxication |
|  |  | 228341007 | Unable to abstain from drinking |
|  |  | 32553006 | Hangover |
|  |  | 228357007 | Persistent effect of alcohol |
|  |  | 228316000 | Alcoholic binges exceeding sensible amounts |
|  |  | 268645007 | Nondependent alcohol abuse |
|  |  | 228354000 | Drink driving |
|  |  | 228317009 | Alcoholic binges exceeding safe amounts |
|  |  | 191883007 | Nondependent alcohol abuse, episodic |
|  |  | 169942003 | Maternal alcohol abuse |
|  |  | 304605000 | Methanol abuse |
|  |  | 288021000119107 | Disorder due to alcohol abuse |
|  |  | 191882002 | Nondependent alcohol abuse, continuous |
|  |  | 15167005 | Alcohol abuse |
|  |  | 284591009 | Persistent alcohol abuse |
|  |  | 228310006 | Drinks in morning to get rid of hangover |
|  |  | 41083005 | Alcohol-induced sleep disorder |
|  |  | 191884001 | Nondependent alcohol abuse in remission |
|  |  | 86325007 | Non megaloblastic anaemia due to alcoholism |
|  |  | 191805002 | Episodic acute alcoholic intoxication in alcoholism |
|  |  | 191802004 | Acute alcoholic intoxication in alcoholism |
|  |  | 7200002 | Alcoholism |
|  |  | 235955000 | Drug-induced chronic pancreatitis |
|  |  | 66590003 | Alcohol dependence |
|  |  | 713583005 | Mild alcohol dependence |
|  |  | 2403008 | Psychoactive substance dependence |
|  |  | 25702006 | Alcohol intoxication |
|  |  | 7200002 | Alcoholism |
|  |  | 308742005 | Alcohol withdrawal-induced convulsion |
|  |  | 713862009 | Severe alcohol dependence |
|  |  | 10755041000119100 | Alcohol dependence in childbirth |
|  |  | 191812006 | Episodic chronic alcoholism |
|  |  | 2403008 | Psychoactive substance dependence |
|  |  | 154211000119108 | Chronic pancreatitis due to chronic alcoholism |
|  |  | 191804003 | Continuous acute alcoholic intoxication in alcoholism |
|  |  | 191813001 | Chronic alcoholism in remission |
|  |  | 7200002 | Alcoholism |
|  |  | 87810006 | Megaloblastic anaemia due to alcoholism |
|  |  | 66590003 | Alcohol dependence |
|  |  | 231467000 | Absinthe addiction |
|  |  | 300939009 | Abstinent alcoholic |
|  |  | 191811004 | Continuous chronic alcoholism |
|  |  | 714829008 | Moderate alcohol dependence |
|  |  | 235952002 | Chronic pancreatitis due to acute alcohol intoxication |
|  |  | 97571000119109 | Thrombocytopenia co-occurrent and due to alcoholism |
|  |  | 66590003 | Alcohol dependence |
|  |  | 10741871000119101 | Alcohol dependence in pregnancy |
|  |  | 288041000119101 | Perceptual disturbance due to alcohol withdrawal |
|  |  | 191480000 | Alcohol withdrawal syndrome |
|  |  | 85561006 | Uncomplicated alcohol withdrawal |
|  |  | 191480000 | Alcohol withdrawal syndrome |
|  |  | 8635005 | Alcohol withdrawal delirium |
|  |  | 79578000 | Alcohol paranoia |
|  |  | 61144001 | Alcohol-induced psychotic disorder with delusions |
|  |  | 191476005 | Alcohol withdrawal hallucinosis |
|  |  | 7052005 | Alcohol hallucinosis |
|  |  | 42344001 | Alcohol-induced psychosis |
|  |  | 191480000 | Alcohol withdrawal syndrome |
|  |  | 191478006 | Alcoholic paranoia |
|  |  | 191471000 | Korsakov's alcoholic psychosis with peripheral neuritis |
|  |  | 73097000 | Alcohol amnestic disorder |
|  |  | 192811002 | Alcoholic encephalopathy |
|  |  | 69482004 | Korsakoff's psychosis |
|  |  | 42344001 | Alcohol-induced psychosis |
|  |  | 281004 | Dementia associated with alcoholism |
|  |  | 231463001 | ^Alcoholic dementia NOS (disorder) |
|  |  | 191475009 | Chronic alcoholic brain syndrome |
|  |  | 78524005 | Alcohol-induced sexual dysfunction |
|  |  | 34938008 | Alcohol-induced anxiety disorder |
|  |  | 228353006 | Reverse tolerance to alcohol |
|  |  | 228351008 | Physical tolerance to alcohol |
|  |  | 228350009 | Behavioural tolerance to alcohol |
|  |  | 53936005 | Alcohol-induced mood disorder |
|  |  | 228323004 | Drinking bout |
|  |  | 29212009 | Alcohol-induced organic mental disorder |
|  |  | 228322009 | Drinking episode |
|  |  | 192206005 | ^Mental and behavioral disorders due to use of alcohol (disorder) |
|  |  | 192207001 | ^Mental and behavioral disorders due to use of alcohol: acute intoxication (disorder) |
|  |  | 192208006 | ^Mental and behavioral disorders due to use of alcohol: harmful use (disorder) |
|  |  | 192209003 | ^Mental and behavioural disorders due to use of alcohol: dependence syndrome) or (chronic alcoholism [& (addiction) or (dipsomania)]) (disorder) |
|  |  | 192210008 | ^Mental and behavioral disorders due to use of alcohol: withdrawal state (disorder) |
|  |  | 192211007 | ^Mental and behavioral disorders due to use of alcohol: withdrawal state with delirium (disorder) |
|  |  | 192212000 | ^Mental and behavioural disorders due to use of alcohol: psychotic disorder (& [hallucinosis] or [jealousy] or [paranoia] or [psychosis NOS] |
|  |  | 192213005 | ^Mental and behavioral disorders due to use of alcohol: amnesic syndrome (disorder) |
|  |  | 192214004 | ^Mental and behavioural disorders due to use of alcohol: residual and late-onset psychotic disorder) or (chronic alcoholic brain syndrome [& dementia NOS] |
|  |  | 192215003 | ^Mental and behavioral disorders due to use of alcohol: other mental and behavioral disorders (disorder) |
|  |  | 268639004 | ^Chronic alcoholism (disorder) |
|  |  | 268683008 | ^Mental and behavioral disorders due to use of alcohol: dependence syndrome (disorder) |
|  |  | 268684002 | ^Mental and behavioral disorders due to use of alcohol: psychotic disorder (disorder) |
|  |  | 304606004 | ^Ethanol abuse (finding) |
|  |  | 268685001 | ^Mental and behavioral disorders due to use of alcohol: residual and late-onset psychotic disorder (disorder) |
|  |  | 192216002 | ^Mental and behavioral disorders due to use of alcohol: unspecified mental and behavioral disorder (disorder) |
| G31.2 | 303 | 192811002 | Alcoholic encephalopathy |
|  |  | 133301000119102 | Degenerative brain disorder due to alcohol |
|  |  | 300992002 | Alcohol-induced cerebellar ataxia |
|  |  | 361272001 | Cerebellar ataxia due to alcoholism |
|  |  | 135761000119101 | Cerebral degeneration due to alcoholism |
|  |  | 230353003 | Morel laminar sclerosis |
|  |  | 361273006 | Alcoholic cerebellar degeneration |
| G62.1 | 357.5 | 192811002 | Alcoholic encephalopathy |
|  |  | 69482004 | Korsakoff's psychosis |
|  |  | 191471000 | Korsakov's alcoholic psychosis with peripheral neuritis |
|  |  | 7916009 | Alcoholic polyneuropathy |
|  |  | 191472007 | #Wernicke-Korsakov syndrome (disorder) |
| G72.1 | *No alcohol-specific code available* | 19303008 | Alcohol myopathy |
| I42.6 | 425.5 | 83521008 | Dilated cardiomyopathy caused by alcohol |
| K29.2 | 535.3 | 2043009 | Alcoholic gastritis |
|  |  | 40241000119109 | Gastric haemorrhage due to alcoholic gastritis |
| K70.0 | 571.0 | 41309000 | Alcoholic liver damage |
| K70.1 | 571.1 | 50325005 | Alcoholic fatty liver |
| K70.2 | 571.2 | 235875008 | Alcoholic hepatitis |
| K70.3 | 571.3 | 9953008 | Acute alcoholic liver disease |
| K70.4 |  | 1085021000119106 | Hepatic ascites due to chronic alcoholic hepatitis |
| K70.9 |  | 1082611000119101 | Ascites due to alcoholic hepatitis |
|  |  | 41309000 | Alcoholic liver damage |
|  |  | 307757001 | Chronic alcoholic hepatitis |
|  |  | 235880004 | Alcoholic fibrosis and sclerosis of liver |
|  |  | 420054005 | Alcoholic cirrhosis |
|  |  | 309783001 | Oesophageal varices in alcoholic cirrhosis of the liver |
|  |  | 1082601000119104 | Ascites due to alcoholic cirrhosis |
|  |  | 235881000 | Alcoholic hepatic failure |
|  |  | 1082621000119108 | Hepatic coma due to alcoholic liver failure |
|  |  | 713370005 | Acute on chronic alcoholic liver disease |
|  |  | 713181003 | Chronic alcoholic liver disease |
| K85.2 | *No alcohol-specific code available* | 235942001 | Alcohol-induced acute pancreatitis |
|  |  | 445507008 | Alcohol-induced pancreatitis |
| K86.0 | *No alcohol-specific code available* | 235952002 | Chronic pancreatitis due to acute alcohol intoxication |
|  |  | 154211000119108 | Chronic pancreatitis due to chronic alcoholism |
| O35.4 | *No alcohol-specific code available* | 199551008 | Maternal care for (suspected) damage to fetus from alcohol |
| P04.3 | 760.71 | 36558000 | Fetal or neonatal effect of alcohol transmitted via placenta and/or breast milk |
|  |  | 268796000 | Fetal or neonatal effect of placental or breast transfer of alcohol |
|  |  | 698321001 | Neonatal effect of alcohol transmitted via breast milk |
|  |  | 205791004 | Fetal or neonatal effect of maternal use of alcohol |
|  |  | 609438005 | Fetal or neonatal effect of maternal alcohol addiction |
| Q86.0 | Alcohol affecting fetus or newborn via placenta or breast milk | 609437000 | Fetal Alcohol Spectrum Disorder |
|  |  | 205788004 | Fetal alcohol syndrome |
|  |  | 205791004 | Fetal or neonatal effect of maternal use of alcohol |
|  |  | 36558000 | Fetal or neonatal effect of alcohol transmitted via placenta and/or breast milk |
| R78.0 | 790.3 | 442766007 | Alcohol in blood specimen above reference range |
|  |  | 442669008 | Ethanol in blood specimen above legal threshold for operating vehicle |
|  |  | 441685000 | Ethanol in blood specimen above reference range |
|  |  | 274776000 | Finding of alcohol in blood |
|  |  | 207273009 | ^Alcohol blood level excessive (situation) |
|  |  | 160592001 | Alcohol intake above recommended sensible limits |
| T51 | 980 | 216633005 | Accidental poisoning by alcoholic beverage |
|  |  | 216635003 | Accidental poisoning by denatured alcohol |
|  |  | 95906008 | Drug interaction with alcohol |
|  |  | 287166006 | Accidental poisoning with ethyl alcohol |
|  |  | 442764005 | Poisoning by benzene |
|  |  | 82782008 | Alcohol poisoning |
|  |  | 212807002 | Grain alcohol causing toxic effect |
|  |  | 216636002 | Accidental poisoning by methylated spirit |
|  |  | 315226008 | Pain in lymph nodes after alcohol consumption |
|  |  | 89507002 | Toxic effect of denatured alcohol |
|  |  | 25966003 | Metabolic acidosis due to methanol |
|  |  | 212809004 | Methyl alcohol causing toxic effect |
|  |  | 216640006 | Accidental poisoning by methanol |
|  |  | 212813006 | Toxic effect of isopropyl alcohol |
|  |  | 6749002 | Toxic effect of propyl alcohol |
|  |  | 216645001 | Accidental poisoning by isopropyl alcohol |
|  |  | 216648004 | Accidental poisoning by rubbing alcohol substitute |
|  |  | 4953006 | Toxic effect of butyl alcohol |
|  |  | 6749002 | Toxic effect of propyl alcohol |
|  |  | 57346004 | Toxic effect of fusel oil |
|  |  | 216651006 | Accidental poisoning by fusel oil |
|  |  | 87460008 | Toxic effect of amyl alcohol |
|  |  | 67426006 | Toxic effect of alcohol |
|  |  | 82047000 | Diarrhoea due to alcohol intake |
|  |  | 314539001 | Alcohol related optic neuropathy |
|  |  | 269765000 | Accidental poisoning by alcohol |
|  |  | 212816003 | ^Rubbing alcohol causing toxic effect (disorder) |
|  |  | 212817007 | ^Isopropyl alcohol causing toxic effect NOS (disorder) |
|  |  | 212818002 | ^Fusel oil causing toxic effect NOS (disorder) |
|  |  | 212819005 | ^Other alcohol causing toxic effect (disorder) |
|  |  | 212820004 | ^Alcohol causing toxic effect NOS (disorder) |
|  |  | 213687005 | ^Toxic effect of other alcohols (disorder) |
|  |  | 212815004 | ^Isopropanol causing toxic effect (disorder) |
|  |  | 212814000 | ^Dimethyl carbinol causing toxic effect (disorder) |
|  |  | 212811008 | ^Wood alcohol causing toxic effect (disorder) |
|  |  | 212808007 | ^Ethyl alcohol causing toxic effect NOS (disorder) |
|  |  | 212806006 | ^Ethyl alcohol causing toxic effect (disorder) |
|  |  | 699208000 | Thrombocytopenia due to alcohol |
| X45 | E860 | 212813006 | Toxic effect of isopropyl alcohol |
|  |  | 216640006 | Accidental poisoning by methanol |
|  |  | 82782008 | Alcohol poisoning |
|  |  | 216635003 | Accidental poisoning by denatured alcohol |
|  |  | 6749002 | Toxic effect of propyl alcohol |
|  |  | 212809004 | Methyl alcohol causing toxic effect |
|  |  | 242263000 | Accidental exposure to alcohol |
|  |  | 216633005 | Accidental poisoning by alcoholic beverage |
|  |  | 212813006 | Toxic effect of isopropyl alcohol |
|  |  | 242265007 | Accidental exposure to ethanol |
|  |  | 278363000 | Alcoholic macrocytosis |
|  |  | 442764005 | Poisoning by benzene |
|  |  | 4953006 | Toxic effect of butyl alcohol |
|  |  | 287166006 | Accidental poisoning with ethyl alcohol |
|  |  | 699208000 | Thrombocytopenia due to alcohol |
|  |  | 212809004 | Methyl alcohol causing toxic effect |
|  |  | 67426006 | Toxic effect of alcohol |
|  |  | 6749002 | Toxic effect of propyl alcohol |
|  |  | 216645001 | Accidental poisoning by isopropyl alcohol |
|  |  | 89507002 | Toxic effect of denatured alcohol |
|  |  | 212807002 | Grain alcohol causing toxic effect |
|  |  | 216648004 | Accidental poisoning by rubbing alcohol substitute |
|  |  | 216651006 | Accidental poisoning by fusel oil |
|  |  | 216636002 | Accidental poisoning by methylated spirit |
|  |  | 89507002 | Toxic effect of denatured alcohol |
|  |  | 442764005 | Poisoning by benzene |
|  |  | 87460008 | Toxic effect of amyl alcohol |
|  |  | 269765000 | Accidental poisoning by alcohol |
|  |  | 57346004 | Toxic effect of fusel oil |
|  |  | 221843007 | ^Accidental poisoning by and exposure to alcohol, occurrence at home (event) |
|  |  | 221844001 | ^Accidental poisoning by and exposure to alcohol, occurrence in residential institution (event) |
|  |  | 221845000 | ^Accidental poisoning by and exposure to alcohol, occurrence at school, other institution and public administrative area (event) |
|  |  | 221846004 | ^Accidental poisoning by and exposure to alcohol, occurrence at sports and athletics area (event) |
|  |  | 221847008 | ^Accidental poisoning by and exposure to alcohol, occurrence on street and highway (event) |
|  |  | 221848003 | ^Accidental poisoning by and exposure to alcohol, occurrence at trade and service area (event) |
|  |  | 221849006 | ^Accidental poisoning by and exposure to alcohol, occurrence at industrial and construction area (event) |
|  |  | 221850006 | ^Accidental poisoning by and exposure to alcohol, occurrence on farm (event) |
|  |  | 221851005 | ^Accidental poisoning by and exposure to alcohol, occurrence at other specified place (event) |
|  |  | 221852003 | ^Accidental poisoning by and exposure to alcohol, occurrence at unspecified place (event) |
|  |  | 57346004 | Toxic effect of fusel oil |
| X65 (*note that no generalised mapping matches were available with SNO-MED although lexical matching suggest use of the above codes*) | *No alcohol-specific code available* | 222103001 | ^Intentional self-poisoning by and exposure to alcohol (event) |
|  |  | 222104007 | ^Intentional self-poisoning by and exposure to alcohol, occurrence at home (event) |
|  |  | 222105008 | ^Intentional self-poisoning by and exposure to alcohol, occurrence in residential institution (event) |
|  |  | 222106009 | ^Intentional self-poisoning by and exposure to alcohol, occurrence at school, other institution and public administrative area (event) |
|  |  | 222107000 | ^Intentional self-poisoning by and exposure to alcohol, occurrence at sports and athletics area (event) |
|  |  | 222108005 | ^Intentional self-poisoning by and exposure to alcohol, occurrence on street and highway (event) |
|  |  | 222110007 | ^Intentional self-poisoning by and exposure to alcohol, occurrence at trade and service area (event) |
|  |  | 222111006 | ^Intentional self-poisoning by and exposure to alcohol, occurrence at industrial and construction area (event) |
|  |  | 222112004 | ^Intentional self-poisoning by and exposure to alcohol, occurrence on farm (event) |
|  |  | 222113009 | ^Intentional self-poisoning by and exposure to alcohol, occurrence at other specified place (event) |
|  |  | 222114003 | ^Intentional self-poisoning by and exposure to alcohol, occurrence at unspecified place (event) |
|  |  | 312963001 | Methanol retinopathy |
| Y15 | E860 | 222702003 | ^Poisoning by and exposure to alcohol, undetermined intent (event) |
|  | 980 | 222703008 | ^Poisoning by and exposure to alcohol, occurrence at home, undetermined intent (event) |
|  |  | 222704002 | ^Poisoning by and exposure to alcohol, occurrence in residential institution, undetermined intent (event) |
|  |  | 222705001 | ^Poisoning by and exposure to alcohol, occurrence at school, other institution and public administrative area, undetermined intent (event) |
|  |  | 222706000 | ^Poisoning by and exposure to alcohol, occurrence at sports and athletics area, undetermined intent (event) |
|  |  | 222707009 | ^Poisoning by and exposure to alcohol, occurrence on street and highway, undetermined intent (event) |
|  |  | 222708004 | ^Poisoning by and exposure to alcohol, occurrence at trade and service area, undetermined intent (event) |
|  |  | 222709007 | ^Poisoning by and exposure to alcohol, occurrence at industrial and construction area, undetermined intent (event) |
|  |  | 222710002 | ^Poisoning by and exposure to alcohol, occurrence on farm, undetermined intent (event) |
|  |  | 222711003 | ^Poisoning by and exposure to alcohol, occurrence at other specified place, undetermined intent (event) |
|  |  | 222713000 | ^Poisoning by and exposure to alcohol, occurrence at unspecified place, undetermined intent (event) |
|  |  | 274776000 | Finding of alcohol in blood |
| Y90 (*note that no generalised mapping matches were available with SNO-MED although lexical matching suggest use of the 'Finding of alcohol in blood' code*) | *No alcohol-specific code available* | 223333005 | ^Evidence of alcohol involvement determined by blood alcohol level (navigational concept) |
|  |  | 223334004 | ^Evidence of alcohol involvement determined by blood alcohol level of less than 20 mg/100 ml (navigational concept) |
|  |  | 223335003 | ^Evidence of alcohol involvement determined by blood alcohol level of 20-39 mg/100 ml (navigational concept) |
|  |  | 223336002 | ^Evidence of alcohol involvement determined by blood alcohol level of 40-59 mg/100 ml (navigational concept) |
|  |  | 223337006 | ^Evidence of alcohol involvement determined by blood alcohol level of 60-79 mg/100 ml (navigational concept) |
|  |  | 223338001 | ^Evidence of alcohol involvement determined by blood alcohol level of 80-99 mg/100 ml (navigational concept) |
|  |  | 223339009 | ^Evidence of alcohol involvement determined by blood alcohol level of 100-119 mg/100 ml (navigational concept) |
|  |  | 223340006 | ^Evidence of alcohol involvement determined by blood alcohol level of 120-199 mg/100 ml (navigational concept) |
|  |  | 223341005 | ^Evidence of alcohol involvement determined by blood alcohol level of 200-239 mg/100 ml (navigational concept) |
|  |  | 223342003 | ^Evidence of alcohol involvement determined by blood alcohol level of 240 mg/100 ml or more (navigational concept) |
|  |  | 223343008 | ^Evidence of alcohol involvement determined by presence of alcohol in blood, level not specified (navigational concept) |
|  |  | 25702006 | Alcohol intoxication |
| Y91 (*note that generalised mapping matches were only available for Y91.1 and Y91.9 with SNO-MED although lexical matching suggest use of 'Alcohol intoxication' code*) | *No alcohol-specific code available* | 230800004 | Alcoholic coma |
|  |  | 82047000 | Diarrhoea due to alcohol intake |
|  |  | 361267005 | Alcohol-related fit |
|  |  | 223344002 | ^Evidence of alcohol involvement determined by level of intoxication (navigational concept) |
|  |  | 223345001 | ^Evidence of alcohol involvement determined by level of intoxication, mild alcohol intoxication (navigational concept) |
|  |  | 223346000 | ^Evidence of alcohol involvement determined by level of intoxication, moderate alcohol intoxication (navigational concept) |
|  |  | 223347009 | ^Evidence of alcohol involvement determined by level of intoxication, severe alcohol intoxication (navigational concept) |
|  |  | 223348004 | ^Evidence of alcohol involvement determined by level of intoxication, very severe alcohol intoxication (navigational concept) |
|  |  | 223349007 | ^Evidence of alcohol involvement determined by level of intoxication, alcohol involvement, not otherwise specified (navigational concept) |

^note that lexical matching suggests use of the above SNOMED codes

# Appendix 2. Cohort formation of The Data-linkage Alcohol Cohort Study (DACS).

The Data-linkage Alcohol Cohort Study (DACS) comprised of NSW residents who had presented to the hospital or emergency department with an alcohol-related problem. Presentations to the hospitals and emergency departments were identified in the NSW Admitted Patient Data Collection (APDC) and Emergency Department Data Collection (EDDC), respectively. Diagnostic codes for an alcohol-related diagnosis were based on the International Classification of Diseases and Health Related Problems, 9th Revision, Clinical Modification (ICD-9-CM), the 10th Revision, Australian Modification (ICD-10-AM) or the Systematized Nomenclature of Medicine--Clinical Terms Australian Modification (SNOMED-CT-AU). Diagnostic codes used are presented in Appendix 1. Individuals who were identified in the NSW APDC and/or EDDC between January 1st 2005 and December 31st 2014 were included in the DACS (see Figure A2).

## NSW Admitted Patient Data Collection (APDC)

The NSW APDC comprised records of hospital separations (including discharges, transfers and deaths) in all public and private hospitals, public multi-purpose services, and day procedure centres in NSW. The records included one field for the principal diagnosis (the condition that is chiefly responsible for occasioning an episode of admitted patient care) and potentially up to 50 secondary diagnoses (conditions that contribute to occasioning an episode of admitted patient care), coded according to ICD-10-AM.

## Emergency Department Data Collection (EDDC)

The NSW EDDC included information about emergency department presentations in major metropolitan and non-metropolitan public hospitals in NSW. Of a total 150 emergency departments that submitted their data to NSW EDDC during the study period, the data captured a substantial proportion of the NSW population although the number of participating emergency departments varies over time. The identification of an alcohol-related presentation was based on the principal diagnosis coded according to ICD-9-CM, ICD-10-AM or SNOMED-CT-AU.

## Cohort inclusion

Individuals who had inconsistent information across datasets (i.e. date of birth or date of death; activities recorded after the date of death; participants with death recorded as the mode of separation from hospital or emergency department but had no mortality record; N=3,545), who were non-NSW residents (N=10,783) or being younger or older than our pre-determined age range (N=389) were excluded. A total of 107 individuals who appeared in the cohort due to prenatal alcohol exposure were also excluded (ICD-10-AM: O35.4, P04.3, Q86.0, and the equivalent ICD-9-CM and SNOMED-CT-AU codes), as these disorders were caused by maternal use of alcohol rather than the individual. A further 4,558 individuals were excluded, who appeared in the cohort due to an indication of alcohol in the blood at their presentations (ICD-10-AM: R78.0, Y90, Y91, and the equivalent ICD-9-CM and SNOMED-CT-AU codes) without any primary code for an alcohol-related diagnosis. These codes were meant to provide supplementary information and not considered as a stand-alone diagnosis. The exclusion groups were non-mutually exclusive. A total of 19,365 (9.3%) individuals were excluded, with the final cohort comprised of 188,778 individuals.

Note that specific to this study, a further 8 participants were excluded as sex was not recorded (see Figure 1 main manuscript).


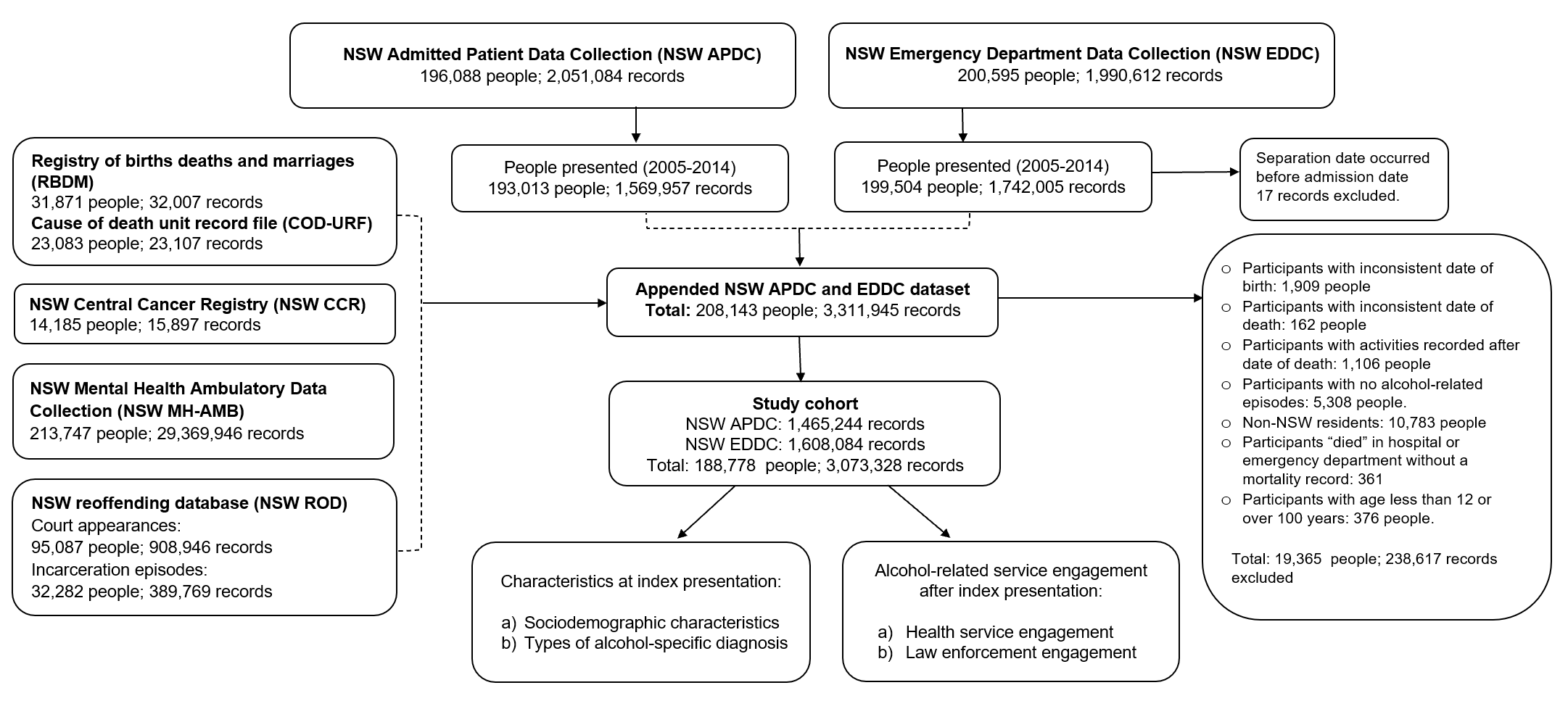


### Figure A2. Formation of The Data-linkage Alcohol Cohort Study (DACS).

Reference: Peacock A, et al. Characterising people attending New South Wales hospitals and emergency departments for an alcohol-related problem: The Data-linkage Alcohol Cohort Study (DACS).

# Appendix 3. Characteristics of individuals first presented to hospital or emergency department with an alcohol-related problem between 2005-2014, and deaths to up until December 31^st^, 2015 (N=188,770)

| **Characteristics at cohort entry** | | **Number of participants (N=188,770)** | **Percent of total** |
| --- | --- | --- | --- |
| Sex |  |  |  |
|  | Male | 125,009 | 66.2% |
|  | Female | 63,761 | 33.8% |
| Age |  |  |  |
|  | Median (IQR) | 39.2 | (24.6, 55.2) |
|  | Mean (SD) | 41.6 | (18.9) |
| Age group | |  |  |
|  | Overall (12+) | 188,770 | 100.0% |
|  | 12-24 | 48,628 | 25.8% |
|  | 25-34 | 32,437 | 17.2% |
|  | 35-44 | 31,763 | 16.8% |
|  | 45-54 | 28,356 | 15.0% |
|  | 55-64 | 21,294 | 11.3% |
|  | 65-74 | 14,922 | 7.9% |
|  | 75-84 | 9,105 | 4.8% |
|  | 85-100 | 2,265 | 1.2% |
| Remoteness of residence | |  |  |
|  | Major cities | 129,428 | 68.9 |
|  | Inner regional | 44,891 | 23.9 |
|  | Outer regional | 12,172 | 6.5 |
|  | Remote | 905 | 0.5 |
|  | Very remote | 365 | 0.2 |
|  | Missing | 1,017 | -- |
| Death status (up to 30 Jun 2016) | |  |  |
|  | Alive | 160,915 | 85.2% |
|  | Dead^a^ | 27,855 | 14.8% |
| Total person-years in the study = 1,079,249;  Average time per person = 5.7 years of follow-up;  Index refers to the event of cohort entry;  Year is defined by calendar years from Jan-Dec;  CI, confidence intervals; IQR, interquartile range; SD, standard deviations.  -- Those with missing data on remoteness of residence were excluded from the computation of percentage of total. | | | |

# Appendix 4. Alcohol-related problem participants presented with at cohort entry


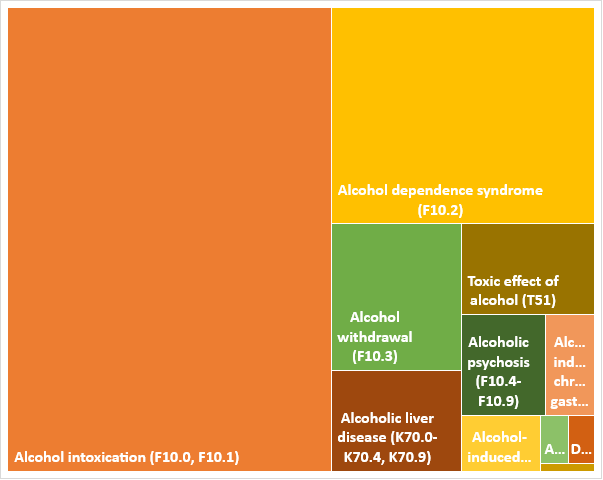


## Figure A4.1. Treemap of alcohol-related problems (ICD-10 codes) participants presented with at cohort entry

*Note.* Participants could present with multiple codes.

## Table A4.1. Alcohol-related problems participants presented with at cohort entry

| **Diagnosis** | **%** |
| --- | --- |
| Toxic effect of alcohol (T51) | 5.0 |
| - Accidental poisoning by and exposure to alcohol (X45) | 0.9 |
| - Intentional self-poisoning by and exposure to alcohol (X65) | 3.5 |
| - Poisoning by and exposure to alcohol, undetermined intent (Y15) | 0.7 |
| Alcohol-induced endocrine and metabolic diseases (E24.4, E51.2) | 0.2 |
| Alcohol intoxication (F10.0, F10.1) | 62.8 |
| Alcohol dependence syndrome (F10.2) | 23.8 |
| Alcohol withdrawal (F10.3) | 8.0 |
| Alcoholic psychosis (F10.4-F10.9) | 3.5 |
| Disease of nervous system due to alcohol (G31.2, G62.1, G72.1) | 0.5 |
| Alcohol cardiomyopathy (I42.6) | 0.6 |
| Alcohol-induced chronic gastritis (K29.2) | 2.1 |
| Alcoholic liver disease (K70.0-K70.4, K70.9) | 5.5 |
| Alcohol-induced pancreatitis (K85.2, K86.0) | 1.8 |

Note. Please see Table A1.1 and Table A1.2 for corresponding ICD-9-CM and SNOMED-CT-AU codes.

# Appendix 5. Causes of deaths attributable to alcohol

We used all APDC diagnosis fields for cohort definition because the aim was to study those presenting with any alcohol-related diagnosis. Studying only those presenting to hospital with an ‘alcohol-induced’ diagnosis (i.e., just those with an alcohol diagnosis in the primary diagnosis field) would significantly narrow the cohort. This approach is consistent with other work (e.g., Sims et al., 2021) and important given evidence of substantial under ascertainment of alcohol-related diagnoses in clinical databases.

We used the underlying and all available contributory causes of death given existing research which has demonstrated the importance of studying both wherever possible so that all available data can be used and underestimation of major causes of death avoided (Batty et al., 2019; Redelings et al., 2006).

**References**

Batty, G. D., Gale, C. R., Kivimäki, M., & Bell, S. (2019). Assessment of relative utility of underlying vs contributory causes of death. JAMA Network Open, 2(7), e198024-e198024.

Redelings, M. D., Sorvillo, F., & Simon, P. (2006). A comparison of underlying cause and multiple causes of death: US vital statistics, 2000–2001. Epidemiology, 17(1), 100-103.

Sims, S., Preen, D., Pereira, G., Fatovich, D., Livingston, M., & O'Donnell, M. (2021). Alcohol-related harm in emergency departments: Linking to subsequent hospitalizations to quantify under-reporting of presentations. Addiction, 116(6), 1371-1380.

## Table A5.1. All causes of deaths fully attributable to alcohol

| **Disease description** | **ICD-10 code** | **Observed deaths** |
| --- | --- | --- |
| **4) Endocrine, nutritional and metabolic diseases** | **E00-E09** |  |
| Alcohol-induced pseudo-Cushing’s syndrome | E24.4 | 0 |
| Wernicke encephalopathy | E51.2 | 42 |
| **5) Mental and behavioural disorders** | **F00-F99** |  |
| Mental and behavioural disorders due to alcohol use | F10 | 2,729 |
| **6) Disease of nervous system** | **G00-G99** |  |
| Degeneration of nervous system due to alcohol | G31.2 | 30 |
| Alcoholic polyneuropathy | G62.1 | <10 |
| Alcoholic myopathy | G72.1 | <10 |
| **9) Diseases of circulatory system** | **I00-I99** |  |
| Alcoholic cardiomyopathy | I42.6 | 174 |
| **11) Diseases of digestive system** | **K00-K93** |  |
| Alcoholic-induced chronic gastritis | K29.2 | <10 |
| Alcoholic liver disease | K70.0-K70.4, K70.9 | 2,434 |
| Alcohol-induced pancreatitis | K85.2, K86.0 | 60 |
| **19) Injury and poisoning** | **S00-T98** |  |
| Alcohol poisoning | T51 | 405 |

## Table A5.2. All-causes of deaths partly attributable to alcohol

| **Cause of death** | **ICD-10 code** | **Observed deaths** |
| --- | --- | --- |
| **1) Certain infectious and parasitic diseases** | **A00-B99** |  |
| Lower respiratory infection: pneumonia | A48.1,A70 | <10 |
| Tuberculosis | A10-A14,A15–A19.9,B90–B90.9 | 19 |
| Viral hepatitis | B15-B19 | 547 |
| **2) Neoplasms** | **C00-D48** |  |
| Breast cancer | C50 | 146 |
| Colon and rectum cancer | C18–C21.9 | 406 |
| Oesophagus cancer | C15–C15.9 | 208 |
| Lip and oral cavity cancer, and pharynx | C00–C08.9 | 187 |
| Liver cancer | C22–C22.9 | 753 |
| **4) Endocrine, nutritional and metabolic diseases** | **E00-E09** |  |
| Diabetes mellitus | E10-E14 | 1,799 |
| **5) Mental and behavioural disorders** | **F00-F99** |  |
| Dementia | F00-F03 | 1,424 |
| Other substance use disorders | F11-F19 | 993 |
| **6) Disease of nervous system** | **G00-G99** |  |
| Alzheimer's disease | G30 | 177 |
| **9) Diseases of circulatory system** | **I00-I99** |  |
| Ischemic heart diseases | I20–I25.9 | 3,887 |
| Stroke | I60-I69.9 | 2,134 |
| Hypertensive heart disease | I11–I11.9 | 128 |
| Cardiomyopathy & myocarditis | I40–I43.9,I51.4–I51.5 | 618 |
| Atrial fibrillation & flutter | I48–I48.92 | 1,063 |
| Heart failure | I50,I11.0,I13.0,I13.2 | 1,829 |
| **10) Diseases of respiratory system** | **J00-J99** |  |
| Chronic obstructive pulmonary disease | J40-J44 | 2,800 |
| Asthma | J45-J46 | 146 |
| **11) Diseases of digestive system** | **K00-K93** |  |
| Cirrhosis & other chronic liver diseases | K71-K77 | 3,994 |
| Diseases of esophagus, stomach and duodenum | K20-K31 | 532 |
| Pancreatic diseases | K85-K87 | 296 |
| **14) Diseases of genitourinary system** | **N00-N99** |  |
| Acute kidney failure and chronic kidney disease | N17-N19 | 2,040 |
| **20) External causes of morbidity and mortality** | **V01-Y98** |  |
| Suicide | X60-X84 | 754 |
| Transport accidents | V01-V99 | 201 |
| Interpersonal violence | X85-Y09 | 79 |

# Appendix 6. ICD-10 diagnostic codes for the disease sub-categories of cause-specific mortality ratios estimated

| **(ICD chapter) Cause of death** | **ICD-10 code** | **Observed deaths** |
| --- | --- | --- |
| **(1) Certain infectious and parasitic diseases** | **A00-B99** | 2,918 |
| Lower respiratory infection: pneumonia | A48.1,A70 | <10† |
| Tuberculosis | A10-A14,A15–A19.9,B90–B90.9 | 19 |
| Viral hepatitis | B15-B19 | 547 |
| **(2) Neoplasms** | **C00-D48** | 5,533 |
| Breast cancer | C50 | 146 |
| Colon and rectal cancer | C18–C21.9 | 406 |
| Oesophageal cancer | C15–C15.9 | 208 |
| Cancer of lip and oral cavity, and pharynx | C00–C08.9, C09–C10.9, C11–C11.9, C32–C32.9, C12–C13.9 | 187 |
| Liver cancer | C22–C22.9 | 753 |
| **(3) Blood and blood-forming organs** | **D50-D89** | 621 |
| **(4) Endocrine, nutritional and metabolic diseases** | **E00-E90** | 2,854 |
| Diabetes mellitus | E10-E14 | 1799 |
| **(5) Mental and behavioural disorders** | **F00-F99** | 5,146 |
| Dementia | F00-F03 | 1424 |
| Alcohol use disorders | F10 | 2729 |
| Other substance use disorders | F11-19 | 993 |
| **(6) Disease of nervous system** | **G00-G99** | 1,360 |
| Alzheimer's disease | G30 | 177 |
| **(7) Eye and adnexa** | **H00-H59** | 41 |
| **(8) Diseases of the ear and mastoid process** | **H60-H95** | <10† |
| **(9) Diseases of circulatory system** | **I00-I99** | 9,876 |
| Ischemic heart diseases | I20–I25.9 | 3887 |
| Stroke | I60-I69.9 | 2134 |
| Hypertensive heart disease | I11–I11.9 | 128 |
| Cardiomyopathy & myocarditis | I40–I43.9,I51.4–I51.5 | 618 |
| Atrial fibrillation & flutter | I48–I48.92 | 1063 |
| Heart failure | I50,I11.0,I13.0,I13.2 | 1829 |
| **(10) Diseases of respiratory system** | **J00-J99** | 6,478 |
| Chronic obstructive pulmonary disease | J40-J44 | 2800 |
| Asthma | J45-J46 | 146 |
| **(11) Diseases of digestive system** | **K00-K93** | 6,365 |
| Cirrhoisis & other chronic liver diseases | K70-K77 | 5273 |
| Diseases of esophagus, stomach and duodenum | K20-K31 | 532 |
| Pancreatic diseases | K85-K87 | 296 |
| **(12) Skin and subcutaneous tissue** | **L00-L99** | 293 |
| **(13) Musculoskeletal system** | **M00-M99** | 635 |
| **(14) Diseases of genitourinary system** | **N00-N99** | 2,349 |
| Acute kidney failure and chronic kidney disease | N17-N19 | 2,040 |
| **(19) Injury and poisoning** | **S00-T98** | 2,986 |
| Alcohol poisoning | T51 | 405 |
| **(20) External causes of morbidity and mortality** | **V01-Y98** | 3,180 |
| Suicide | X60-X84 | 754 |
| Transport accidents | V01-V99 | 201 |
| Interpersonal violence | X85-Y09 | 79 |

*Note.* ICD chapters 15, 16 and 17 are excluded because they are primarily conditions related to pregnancy, childbirth and puerperium (15), or perinatal (16) and congenital (17) conditions. ICD chapter 18 is excluded because it generally relates to non-specific findings.

† Categories with number of deaths <10 are excluded from analysis.

## References

**National Alcohol Indicators (Feb 2018)**

<https://ndri.curtin.edu.au/ndri/media/documents/naip/naip016.pdf>

**ICD-10 mapping**

<https://espace.curtin.edu.au/bitstream/handle/20.500.11937/34117/19233_downloaded_stream_325.pdf>

**Literature**

Askgaard, G. et. al. (2019) Hospital admissions and mortality in the 15 years after a first-time hospital contact with an alcohol problem: a prospective cohort study using the entire Danish population.

Rehm J. et. al. (2016) The relationship between different dimensions of alcohol use and the burden of disease - an update.

# Appendix 7. Count regression models for analysis of excess mortality

## Table A7.1 Overdispersion parameter in the negative binomial models for analysis of excess mortality

| **Model** | **Overall (Total)** | | **By sex** | |
| --- | --- | --- | --- | --- |
|  | **Est. (95% CI)** | **χ^2^ (p)**† | **Est. (95% CI)** | **χ^2^ (p)**† |
| All-cause mortality | 0.25 (0.15, 0.42) | **3412 (<0.001)** | 0.24 (0.14, 0.41) | **3412 (<0.001)** |
| All-cause mortality by age | 0.03 (0.01, 0.05) | **281 (<0.001)** | 0.01 (0.01, 0.02) | **130 (<0.001)** |
| **Attribution to alcohol†** |  |  |  |  |
| All causes *fully* attributable to alcohol | 0.55 (0.33, 0.90) | **972 (<0.001)** | 0.34 (0.19, 0.59) | **400 (<0.001)** |
| All causes *fully or partly* attributable to alcohol | 0.27 (0.16, 0.44) | **3456 (<0.001)** | 0.26 (0.16, 0.43) | **3447 (<0.001)** |
| **Either fully or partly attributable to alcohol** | 0.31 (0.19, 0.51) | **4277 (<0.001)** | 0.30 (0.18, 0.49) | **4275 (<0.001)** |
| **1) Certain infectious and parasitic diseases (A00-B99)** | 0.64 (0.36, 1.11) | **1056 (<0.001)** | 0.62 (0.35, 1.09) | **1056 (<0.001)** |
| Tuberculosis | 0.00 (--) | <0.1 (0.500) | 0.00 (--) | <0.1 (0.500) |
| Viral hepatitis | 0.20 (0.08, 0.51) | **24.1 (<0.001)** | 0.11 (0.03, 0.35) | **15.7 (0.000)** |
| **2) Neoplasms (C00-D48)** | 0.17 (0.08, 0.34) | **398 (<0.001)** | 0.15 (0.08, 0.32) | **364 (<0.001)** |
| Breast cancer | 0.19 (0.05, 0.72) | **8.6 (0.002)** | 0.19 (0.05, 0.71) | **8.6 (0.002)** |
| Colon and rectal cancer | 0.00 (--) | <0.1 (0.434) | 0.00 (--) | <0.1 (0.499) |
| Oesophageal cancer | 0.24 (0.07, 0.82) | **11.6 (<0.001)** | 0.16 (0.04, 0.57) | **9.0 (0.001)** |
| Cancer of lip and oral cavity, and pharynx | 0.17 (0.04, 0.71) | **6.4 (0.006)** | 0.16 (0.04, 0.69) | **6.2 (0.006)** |
| Liver cancer | 0.27 (0.12, 0.61) | **78.6 (<0.001)** | 0.19 (0.08, 0.49) | **60.2 (<0.001)** |
| **3) Blood and blood-forming organs (D50-D89)** | 0.44 (0.23, 0.84) | **171 (<0.001)** | 0.43 (0.23, 0.82) | **170 (<0.001)** |
| **4) Endocrine, nutritional and metabolic diseases (E00-E90)** | 0.28 (0.16, 0.49) | **542 (<0.001)** | 0.28 (0.16, 0.49) | **521 (<0.001)** |
| Diabetes mellitus | 0.34 (0.19, 0.61) | **380 (<0.001)** | 0.33 (0.18, 0.61) | **351 (<0.001)** |
| **5) Mental and behavioural disorders (F00-F99)** | 0.48 (0.29, 0.77) | **2018 (<0.001)** | 0.42 (0.26, 0.69) | **2016 (<0.001)** |
| Dementia | 0.16 (0.07, 0.36) | **106 (<0.001)** | 0.16 (0.07, 0.36) | **92.1 (<0.001)** |
| Alcohol use disorders | 0.58 (0.35, 0.96) | **532 (<0.001)** | 0.35 (0.20, 0.64) | **201 (<0.001)** |
| Other substance use disorders | 0.20 (0.11, 0.37) | **116 (<0.001)** | 0.10 (0.05, 0.21) | **55.8 (<0.001)** |
| **6) Disease of nervous system (G00-G99)** | 0.28 (0.16, 0.49) | **298 (<0.001)** | 0.27 (0.15, 0.48) | **294 (<0.001)** |
| Alzheimer's disease | 0.00 (--) | <0.1 (0.500) | 0.00 (--) | <0.1 (0.499) |
| **7) Diseases of the eye and adnexa (H00-H59)** | 0.16 (0.01, 1.82) | 1.1 (0.147) | 0.16 (0.01, 2.21) | 0.9 (0.172) |
| **9) Diseases of circulatory system (I00-I99)** | 0.23 (0.13, 0.40) | **1478 (<0.001)** | 0.22 (0.13, 0.39) | **1451 (<0.001)** |
| Ischemic heart diseases | 0.20 (0.11, 0.35) | **451 (<0.001)** | 0.19 (0.11, 0.33) | **453 (<0.001)** |
| Stroke | 0.20 (0.11, 0.36) | **344 (<0.001)** | 0.20 (0.11, 0.36) | **318 (<0.001)** |
| Hypertensive heart disease | 0.17 (0.05, 0.65) | **6.7 (0.005)** | 0.17 (0.04, 0.69) | **5.9 (0.008)** |
| Cardiomyopathy & myocarditis | 0.24 (0.10, 0.54) | **48.5 (<0.001)** | 0.23 (0.10, 0.54) | **43.4 (<0.001)** |
| Atrial fibrillation & flutter | 0.19 (0.09, 0.41) | **76.5 (<0.001)** | 0.16 (0.07, 0.35) | **64.0 (<0.001)** |
| Heart failure | 0.24 (0.13, 0.45) | **250 (<0.001)** | 0.24 (0.13, 0.45) | **237 (<0.001)** |
| **10) Diseases of respiratory system (J00-J99)** | 0.31 (0.17, 0.54) | **973 (<0.001)** | 0.29 (0.16, 0.52) | **968 (<0.001)** |
| Chronic obstructive pulmonary disease | 0.25 (0.13, 0.48) | **461 (<0.001)** | 0.23 (0.12, 0.44) | **400 (<0.001)** |
| Asthma | 0.35 (0.12, 0.99) | **12.5 (<0.001)** | 0.35 (0.12, 0.99) | **12.6 (<0.001)** |
| **11) Diseases of digestive system (K00-K93)** | 0.56 (0.33, 0.97) | **1917 (<0.001)** | 0.54 (0.32, 0.94) | **1918 (<0.001)** |
| Cirrhosis & other chronic liver diseases | 0.17 (0.09, 0.32) | **297 (<0.001)** | 0.12 (0.06, 0.23) | **176 (<0.001)** |
| Esophagus, stomach and duodenum | 0.51 (0.27, 0.94) | **176 (<0.001)** | 0.51 (0.27, 0.93) | **174 (<0.001)** |
| Pancreatic diseases | 0.33 (0.16, 0.70) | **47.4 (<0.001)** | 0.32 (0.15, 0.67) | **47.2 (<0.001)** |
| **12) Skin and subcutaneous tissue (L00-L99)** | 0.36 (0.17, 0.75) | **54.6 (<0.001)** | 0.36 (0.17, 0.75) | **53.9 (<0.001)** |
| **13) Musculoskeletal system (M00-M99)** | 0.19 (0.09, 0.43) | **45.6 (<0.001)** | 0.19 (0.08, 0.42) | **43.1 (<0.001)** |
| **14) Diseases of genitourinary system (N00-N99)** | 0.34 (0.19, 0.62) | **550 (<0.001)** | 0.34 (0.19, 0.62) | **547 (<0.001)** |
| Acute kidney failure and chronic kidney disease | 0.38 (0.21, 0.68) | **501 (<0.001)** | 0.38 (0.21, 0.68) | **498 (<0.001)** |
| **19)** **Injury and poisoning (S00-T98)** | 0.38 (0.23, 0.63) | **601 (<0.001)** | 0.29 (0.17, 0.48) | **493 (<0.001)** |
| Alcohol poisoning | 0.20 (0.09, 0.46) | **31.3 (<0.001)** | 0.01 (0.00, 0.43) | <0.1 (0.259) |
| **20) External causes of morbidity and mortality (V01-Y98)** | 0.35 (0.21, 0.58) | **612 (<0.001)** | 0.27 (0.16, 0.45) | **516 (<0.001)** |
| Suicide | 0.36 (0.19, 0.67) | **90.3 (<0.001)** | 0.10 (0.04, 0.24) | **23.2 (<0.001)** |
| Transport accidents | 0.09 (0.02, 0.36) | **5.1 (0.012)** | 0.09 (0.02, 0.36) | **5.0 (0.013)** |
| Interpersonal violence | 0.17 (0.03, 0.90) | **2.8 (0.046)** | 0.10 (0.01, 0.98) | 1.3 (0.125) |
| † Chi-square test for null hypothesis: dispersion parameter = 0. Statistics showing significant difference of dispersion parameter from 0 at p<0.05 are highlighted in bold. | | | | |

## Table A7.2 Total - Estimated overall standardised mortality ratio (SMR) in the Poisson and negative binomial models

| **Model** | **Poisson** | **Negative binomial** |
| --- | --- | --- |
|  | **Overall SMR (95% CI)** | **Overall SMR (95% CI)** |
| All-cause mortality | 6.2 (6.1, 6.3) | 6.2 (5.4, 7.2)* |
| All-cause mortality by age |  |  |
| 12-24 | 2.5 (2.2, 2.8) | 2.5 (1.8, 3.5)* |
| 25-34 | 6.8 (6.4, 7.3) | 6.7 (5.6, 8.0)* |
| 35-44 | 8.5 (8.2, 8.9) | 8.4 (7.4, 9.6)* |
| 45-54 | 10.0 (9.7, 10.3) | 10.0 (9.1, 10.9)* |
| 55-64 | 8.8 (8.5, 9.0) | 8.7 (7.8, 9.8)* |
| 65-74 | 6.7 (6.6, 6.9) | 6.7 (6.0, 7.5)* |
| 75-84 | 4.6 (4.5, 4.7) | 4.6 (4.3, 4.9)* |
| 85+ | 4.0 (3.8, 4.1) | 4.0 (3.5, 4.6)* |
| **Attribution to alcohol†** |  |  |
| All causes *fully* attributable to alcohol | 51.0 (49.6, 52.4) | 50.3 (44.6, 56.8)* |
| All causes *fully or partly* attributable to alcohol | 7.5 (7.4, 7.6) | 7.5 (6.1, 9.3)* |
| **Either fully or partly attributable to alcohol** | 8.0 (7.9, 8.1) | 8.0 (6.4, 10.0)* |
| **1) Certain infectious and parasitic diseases (A00-B99)** | 11.3 (10.9, 11.7) | 11.2 (8.3, 15.1)* |
| Tuberculosis | 5.6 (3.6, 8.8)* | 5.6 (4.2, 7.4) |
| Viral hepatitis | 28.5 (26.2, 30.9) | 29.4 (24.6, 35.2)* |
| **2) Neoplasms (C00-D48)** | 4.8 (4.6, 4.9) | 4.8 (4.2, 5.5)* |
| Breast cancer | 3.1 (2.7, 3.7) | 3.2 (2.4, 4.2)* |
| Colon and rectal cancer | 3.7 (3.3, 4.0)* | 4.0 (3.7, 4.3) |
| Oesophageal cancer | 5.7 (4.9, 6.5) | 5.3 (4.2, 6.7)* |
| Cancer of lip and oral cavity, and pharynx | 13.4 (11.6, 15.5) | 13.2 (11.0, 15.8)* |
| Liver cancer | 17.7 (16.5, 19.0) | 18.3 (14.8, 22.5)* |
| **3) Blood and blood-forming organs (D50-D89)** | 7.6 (7.0, 8.2) | 7.3 (5.5, 9.8)* |
| **4) Endocrine, nutritional and metabolic diseases (E00-E90)** | 6.5 (6.2, 6.7) | 6.4 (5.1, 8.0)* |
| Diabetes mellitus | 5.9 (5.7, 6.2) | 5.8 (4.6, 7.4)* |
| **5) Mental and behavioural disorders (F00-F99)** | 13.1 (12.8, 13.5) | 13.0 (9.9, 17.2)* |
| Dementia | 6.4 (6.1, 6.8) | 6.2 (5.3, 7.3)* |
| Alcohol use disorders | 47.8 (46.1, 49.6) | 46.7 (41.4, 52.7)* |
| Other substance use disorders | 13.8 (13.0, 14.7) | 13.2 (10.9, 15.9)* |
| **6) Disease of nervous system (G00-G99)** | 5.1 (4.9, 5.4) | 5.0 (3.9, 6.5)* |
| Alzheimer's disease | 3.3 (2.8, 3.8)* | 3.3 (3.1, 3.6) |
| **7) Diseases of the eye and adnexa (H00-H59)** | 5.1 (3.8, 6.9)* | 4.5 (3.0, 6.6) |
| **9) Diseases of circulatory system (I00-I99)** | 6.2 (6.1, 6.3) | 6.2 (5.1, 7.4)* |
| Ischemic heart diseases | 5.2 (5.0, 5.4) | 5.1 (4.3, 6.1)* |
| Stroke | 5.9 (5.6, 6.1) | 5.8 (4.7, 7.2)* |
| Hypertensive heart disease | 5.7 (4.8, 6.8) | 5.5 (4.1, 7.4)* |
| Cardiomyopathy & myocarditis | 11.1 (10.3, 12.0) | 11.3 (9.3, 13.6)* |
| Atrial fibrillation & flutter | 6.2 (5.8, 6.6) | 6.0 (5.2, 6.9)* |
| Heart failure | 5.9 (5.7, 6.2) | 5.7 (4.8, 6.9)* |
| **10) Diseases of respiratory system (J00-J99)** | 7.5 (7.3, 7.7) | 7.4 (6.2, 8.9)* |
| Chronic obstructive pulmonary disease | 9.7 (9.4, 10.1) | 9.6 (7.7, 12.0)* |
| Asthma | 5.8 (4.9, 6.8) | 5.6 (4.4, 7.0)* |
| **11) Diseases of digestive system (K00-K93)** | 21.2 (20.7, 21.7) | 21.2 (16.5, 27.2)* |
| Cirrhosis & other chronic liver diseases | 39.2 (38.1, 40.2) | 39.0 (35.5, 42.9)* |
| Esophagus, stomach and duodenum | 12.1 (11.2, 13.2) | 11.7 (8.4, 16.4)* |
| Pancreatic diseases | 24.9 (22.2, 27.9) | 23.8 (17.9, 31.5)* |
| **12) Skin and subcutaneous tissue (L00-L99)** | 8.3 (7.4, 9.3) | 7.8 (5.8, 10.5)* |
| **13) Musculoskeletal system (M00-M99)** | 6.2 (5.8, 6.8) | 5.8 (5.0, 6.8)* |
| **14) Diseases of genitourinary system (N00-N99)** | 6.5 (6.2, 6.8) | 6.3 (5.0, 8.0)* |
| Acute kidney failure and chronic kidney disease | 6.6 (6.4, 6.9) | 6.5 (5.1, 8.3)* |
| **19)** **Injury and poisoning (S00-T98)** | 9.3 (9.0, 9.7) | 9.2 (7.9, 10.8)* |
| Alcohol poisoning | 29.4 (26.7, 32.4) | 26.0 (21.1, 32.0*) |
| **20) External causes of morbidity and mortality (V01-Y98)** | 8.9 (8.6, 9.2) | 8.8 (7.5, 10.3)* |
| Suicide | 9.7 (9.0, 10.4) | 9.1 (8.1, 10.3)* |
| Transport accidents | 4.0 (3.5, 4.6) | 3.8 (3.1, 4.8)* |
| Interpersonal violence | 8.8 (7.0, 10.9) | 7.9 (5.7, 11.1)* |
| *** Chosen model for presenting in Tables 2 and 4 – Negative binomial model is used where there was significant overdispersion as shown in Table A7.1.** | | |

## Table A7.3 By sex - Estimated standardised mortality ratio (SMR) and relative risk (RR) of sex in the Poisson and negative binomial models

| **Model** | **Poisson** | | | **Negative binomial** | | |
| --- | --- | --- | --- | --- | --- | --- |
|  | **SMR (95% CI)** | | **RR (95%CI)** | **SMR (95% CI)** | | **RR (95%CI)** |
|  | **Males** | **Females** | **Females (ref: Males)** | **Males** | **Females** | **Females (ref: Males)** |
| All-cause mortality | 6.2 (6.1, 6.3) | 6.2 (6.0, 6.3) | 1.0 (1.0, 1.0) | 6.2 (5.2, 7.4)* | 6.1 (4.6, 8.2)* | 1.0 (0.7, 1.4)* |
| All-cause mortality by age |  |  |  |  |  |  |
| 12-24 | 2.4 (2.1, 2.7) | 2.8 (2.2, 3.5) | 1.2 (0.9, 1.5) | 2.5 (1.7, 3.6)* | 2.8 (1.4, 5.8)* | 1.1 (0.5, 2.6)* |
| 25-34 | 6.4 (5.9, 6.9) | 9.0 (7.8, 10.4) | 1.4 (1.2, 1.7) | 6.4 (5.3, 7.7)* | 9.1 (7.4, 11.2)* | 1.4 (1.1, 1.9)* |
| 35-44 | 8.0 (7.6, 8.4) | 10.5 (9.6, 11.4) | 1.3 (1.2, 1.5) | 8.0 (7.0, 9.2)* | 10.5 (10.1, 10.8)* | 1.3 (1.1, 1.5)* |
| 45-54 | 9.7 (9.4, 10.1) | 10.9 (10.3, 11.6) | 1.1 (1.0, 1.2) | 9.7 (8.8, 10.8)* | 10.9 (9.8, 12.3)* | 1.1 (1.0, 1.3)* |
| 55-64 | 8.5 (8.2, 8.7) | 10.2 (9.6, 10.8) | 1.2 (1.1, 1.3) | 8.5 (7.5, 9.5)* | 10.2 (9.9, 10.6)* | 1.2 (1.1, 1.4)* |
| 65-74 | 6.5 (6.3, 6.7) | 7.9 (7.5, 8.4) | 1.2 (1.1, 1.3) | 6.5 (5.8, 7.3)* | 7.9 (6.7, 9.3)* | 1.2 (1.0, 1.5)* |
| 75-84 | 4.7 (4.5, 4.8) | 4.2 (4.0, 4.5) | 0.9 (0.9, 1.0) | 4.7 (4.4, 5.0)* | 4.2 (3.4, 5.2)* | 0.9 (0.7, 1.1)* |
| 85+ | 4.3 (4.2, 4.5) | 3.4 (3.2, 3.6) | 0.8 (0.7, 0.8) | 4.3 (3.9, 4.8)* | 3.3 (2.8, 4.0)* | 0.8 (0.6, 0.9)* |
| **Attribution to alcohol†** |  |  |  |  |  |  |
| All causes *fully* attributable to alcohol | 44.6 (43.3, 46.0) | 110.0 (103.7, 116.8) | 2.5 (2.3, 2.6) | 44.7 (40.2, 49.7)* | 110.1 (90.7, 133.6)* | 2.5 (2.0, 3.1)* |
| All causes *fully or partly* attributable to alcohol | 7.6 (7.5, 7.8) | 7.2 (6.9, 7.4) | 0.9 (0.9, 1.0) | 7.6 (6.0, 9.8)* | 7.1 (4.9, 10.3)* | 0.9 (0.6, 1.5)* |
| **Either fully or partly attributable to alcohol** | 8.1 (7.9, 8.2) | 7.8 (7.6, 8.1) | 1.0 (0.9, 1.0) | 8.1 (6.2, 10.5)* | 7.7 (5.2, 11.5)* | 1.0 (0.6, 1.5)* |
| **1) Certain infectious and parasitic diseases (A00-B99)** | 11.3 (10.9, 11.8) | 11.1 (10.2, 12.0) | 1.0 (0.9, 1.1) | 11.3 (8.0, 16.0)* | 10.8 (6.6, 17.7)* | 1.0 (0.5, 1.7)* |
| Tuberculosis | 5.9 (3.7, 9.6)* | -- | 0.7 (0.2, 2.8)* | 5.7 (4.3, 7.6) | -- | 0.7 (0.2, 2.3) |
| Viral hepatitis | 26.6 (24.3, 29.2) | 42.0 (34.4, 51.2) | 1.6 (1.3, 2.0) | 29.2 (22.5, 37.8)* | 49.2 (41.4, 58.4)* | 1.7 (1.3, 2.1)* |
| **2) Neoplasms (C00-D48)** | 4.9 (4.8, 5.1) | 4.0 (3.8, 4.3) | 0.8 (0.8, 0.9) | 5.0 (4.2, 5.9)* | 4.1 (3.5, 4.7)* | 0.8 (0.7, 1.0)* |
| Breast cancer | -- | 3.1 (2.6, 3.7) | 0.7 (0.3, 1.7) | -- | 3.2 (2.4, 4.2)* | 0.6 (0.3, 1.6)* |
| Colon and rectal cancer | 3.9 (3.5, 4.3)* | 2.7 (2.1, 3.5)* | 0.7 (0.5, 0.9)* | 4.0 (3.7, 4.3) | 2.7 (1.9, 3.7) | 0.7 (0.5, 0.9) |
| Oesophageal cancer | 5.2 (4.4, 6.0) | 9.8 (7.1, 13.4) | 1.9 (1.3, 2.7) | 5.2 (4.1, 6.7)* | 8.0 (4.4, 14.5)* | 1.5 (0.8, 2.9)* |
| Cancer of lip and oral cavity, and pharynx | 13.1 (11.2, 15.3) | 15.6 (10.7, 22.8) | 1.2 (0.8, 1.8) | 13.1 (10.9, 15.9)* | 15.4 (9.5, 24.9)* | 1.2 (0.7, 2.0)* |
| Liver cancer | 18.8 (17.4, 20.2) | 10.2 (7.9, 13.3) | 0.5 (0.4, 0.7) | 19.0 (15.3, 23.6)* | 10.0 (6.5, 15.4)* | 0.5 (0.3, 0.9)* |
| **3) Blood and blood-forming organs (D50-D89)** | 7.4 (6.7, 8.1) | 8.3 (7.1, 9.6) | 1.1 (0.9, 1.3) | 7.3 (5.2, 10.1)* | 7.7 (4.5, 13.2)* | 1.1 (0.6, 2.0)* |
| **4) Endocrine, nutritional and metabolic diseases (E00-E90)** | 6.8 (6.5, 7.0) | 5.4 (5.0, 5.9) | 0.8 (0.7, 0.9) | 6.7 (5.2, 8.6)* | 5.1 (3.5, 7.6)* | 0.8 (0.5, 1.2)* |
| Diabetes mellitus | 6.3 (6.0, 6.6) | 4.5 (4.0, 5.0) | 0.7 (0.6, 0.8) | 6.2 (4.8, 8.1)* | 4.0 (2.5, 6.2)* | 0.6 (0.4, 1.1)* |
| **5) Mental and behavioural disorders (F00-F99)** | 13.4 (13.0, 13.8) | 12.4 (11.7, 13.1) | 0.9 (0.9, 1.0) | 13.4 (9.7, 18.4)* | 12.0 (6.9, 20.6)* | 0.9 (0.5, 1.7)* |
| Dementia | 6.9 (6.5, 7.3) | 5.5 (5.0, 6.1) | 0.8 (0.7, 0.9) | 6.7 (5.5, 8.1)* | 5.2 (4.2, 6.4)* | 0.8 (0.6, 1.0)* |
| Alcohol use disorders | 41.8 (40.0, 43.5) | 111.0 (102.2, 120.7) | 2.7 (2.4, 2.9) | 41.9 (37.7, 46.5)* | 112.0 (92.9, 135.1)* | 2.7 (2.2, 3.3)* |
| Other substance use disorders | 12.1 (11.3, 13.0) | 23.7 (20.9, 26.8) | 2.0 (1.7, 2.3) | 12.0 (9.9, 14.5)* | 23.4 (18.3, 30.1)* | 2.0 (1.4, 2.7)* |
| **6) Disease of nervous system (G00-G99)** | 5.3 (5.0, 5.6) | 4.6 (4.1, 5.2) | 0.9 (0.8, 1.0) | 5.2 (3.8, 7.1)* | 4.4 (3.2, 5.9)* | 0.8 (0.5, 1.3)* |
| Alzheimer's disease | 3.4 (2.8, 4.1) | 3.1 (2.4, 4.0) | 0.9 (0.7, 1.3) | 3.4 (3.3, 3.5)* | 3.2 (2.5, 4.0)* | 0.9 (0.7, 1.2)* |
| **7) Diseases of the eye and adnexa (H00-H59)** | 4.8 (3.3, 7.1) | 5.6 (3.4, 9.3) | 1.2 (0.6, 2.2) | 3.8 (2.5, 5.9)* | 6.0 (3.6, 10.1)* | 1.6 (0.8, 3.1)* |
| **9) Diseases of circulatory system (I00-I99)** | 6.4 (6.2, 6.5) | 5.6 (5.3, 5.8) | 0.9 (0.8, 0.9) | 6.3 (5.1, 7.9)* | 5.5 (3.9, 7.6)* | 0.9 (0.6, 1.3)* |
| Ischemic heart diseases | 5.2 (5.1, 5.4) | 5.0 (4.7, 5.4) | 1.0 (0.9, 1.0) | 5.2 (4.3, 6.3)* | 4.6 (3.2, 6.6)* | 0.9 (0.6, 1.3)* |
| Stroke | 6.3 (6.0, 6.6) | 4.8 (4.4, 5.3) | 0.8 (0.7, 0.9) | 6.2 (4.8, 8.0)* | 4.5 (3.1, 6.4)* | 0.7 (0.5, 1.1)* |
| Hypertensive heart disease | 6.0 (4.9, 7.2) | 4.9 (3.3, 7.2) | 0.8 (0.5, 1.3) | 5.9 (4.4, 8.0)* | 3.2 (1.9, 5.4)* | 0.5 (0.3, 1.0)* |
| Cardiomyopathy & myocarditis | 11.5 (10.6, 12.5) | 8.7 (7.0, 10.9) | 0.8 (0.6, 1.0) | 11.6 (9.5, 14.1)* | 7.7 (4.5, 13.0)* | 0.7 (0.4, 1.2)* |
| Atrial fibrillation & flutter | 6.6 (6.2, 7.1) | 5.0 (4.4, 5.7) | 0.8 (0.6, 0.9) | 6.3 (5.3, 7.5)* | 4.6 (3.6, 5.9)* | 0.7 (0.5, 1.0)* |
| Heart failure | 6.2 (5.9, 6.6) | 5.1 (4.6, 5.6) | 0.8 (0.7, 0.9) | 6.1 (4.9, 7.5)* | 4.5 (3.4, 5.9)* | 0.7 (0.5, 1.0)* |
| **10) Diseases of respiratory system (J00-J99)** | 7.4 (7.2, 7.6) | 8.0 (7.6, 8.4) | 1.1 (1.0, 1.1) | 7.3 (5.9, 9.1)* | 7.8 (5.6, 10.9)* | 1.1 (0.7, 1.6)* |
| Chronic obstructive pulmonary disease | 9.0 (8.7, 9.4) | 13.0 (12.1, 14.0) | 1.4 (1.3, 1.6) | 8.9 (7.0, 11.4)* | 12.7 (9.5, 17.0)* | 1.4 (1.0, 2.1)* |
| Asthma | 5.9 (4.8, 7.2) | 5.7 (4.3, 7.5) | 1.0 (0.7, 1.4) | 5.8 (4.5, 7.5)* | 5.0 (3.0, 8.2)* | 0.9 (0.5, 1.5)* |
| **11) Diseases of digestive system (K00-K93)** | 21.2 (20.6, 21.8) | 21.2 (20.1, 22.4) | 1.0 (0.9, 1.1) | 21.2 (16.0, 28.2)* | 21.1 (13.0, 34.2)* | 1.0 (0.6, 1.7)* |
| Cirrhosis & other chronic liver diseases | 36.5 (35.4, 37.6) | 54.6 (51.4, 57.9) | 1.5 (1.4, 1.6) | 36.7 (33.4, 40.5)* | 56.1 (45.9, 68.5)* | 1.5 (1.2, 1.9)* |
| Esophagus, stomach and duodenum | 12.6 (11.4, 13.8) | 10.5 (8.6, 12.8) | 0.8 (0.7, 1.0) | 12.4 (8.5, 18.0)* | 8.3 (4.4, 15.7)* | 0.7 (0.3, 1.4)* |
| Pancreatic diseases | 24.0 (21.1, 27.4) | 28.4 (22.5, 35.9) | 1.2 (0.9, 1.5) | 23.7 (17.4, 32.2)* | 24.4 (12.7, 47.0)* | 1.0 (0.5, 2.1)* |
| **12) Skin and subcutaneous tissue (L00-L99)** | 8.6 (7.5, 9.8) | 7.7 (6.1, 9.6) | 0.9 (0.7, 1.2) | 8.2 (5.7, 11.7)* | 6.3 (4.4, 9.0)* | 0.8 (0.5, 1.2)* |
| **13) Musculoskeletal system (M00-M99)** | 6.5 (5.9, 7.2) | 5.7 (5.0, 6.6) | 0.9 (0.7, 1.0) | 6.1 (5.1, 7.4)* | 4.9 (3.7, 6.4)* | 0.8 (0.6, 1.0)* |
| **14) Diseases of genitourinary system (N00-N99)** | 6.6 (6.3, 6.9) | 6.0 (5.5, 6.6) | 0.9 (0.8, 1.0) | 6.5 (4.9, 8.6)* | 5.6 (3.9, 8.2)* | 0.9 (0.5, 1.4)* |
| Acute kidney failure and chronic kidney disease | 6.8 (6.5, 7.1) | 6.1 (5.5, 6.7) | 0.9 (0.8, 1.0) | 6.7 (5.0, 8.9)* | 5.6 (3.8, 8.3)* | 0.8 (0.5, 1.3)* |
| **19)** **Injury and poisoning (S00-T98)** | 8.4 (8.1, 8.8) | 13.5 (12.6, 14.5) | 1.6 (1.5, 1.7) | 8.4 (7.2, 9.9)* | 13.4 (9.2, 19.5)* | 1.6 (1.0, 2.4)* |
| Alcohol poisoning | 24.3 (21.7, 27.3)* | 58.2 (48.7, 69.6)* | 2.4 (1.9, 3.0)* | 23.7 (20.6, 27.3) | 57.5 (50.4, 65.6) | 2.4 (2.0, 2.9) |
| **20) External causes of morbidity and mortality (V01-Y98)** | 8.1 (7.8, 8.4) | 12.5 (11.7, 13.4) | 1.5 (1.4, 1.7) | 8.1 (6.9, 9.5)* | 12.3 (8.5, 17.9)* | 1.5 (1.0, 2.3)* |
| Suicide | 8.2 (7.5, 8.9) | 19.2 (16.7, 22.0) | 2.3 (2.0, 2.7) | 8.5 (7.5, 9.6)* | 18.7 (13.7, 25.5)* | 2.2 (1.6, 3.1)* |
| Transport accidents | 4.0 (3.5, 4.7) | 4.2 (2.9, 6.1) | 1.1 (0.7, 1.6) | 3.8 (3.0, 4.8)* | 3.8 (2.0, 7.1)* | 1.0 (0.5, 1.9)* |
| Interpersonal violence | 7.7 (5.9, 10.0)* | 13.6 (8.9, 20.6)* | 1.8 (1.1, 2.9)* | 7.4 (5.3, 10.5) | 14.1 (7.9, 25.1) | 1.9 (1.0, 3.7) |
| *** Chosen model for presenting in Tables 2 and 4 – Negative binomial model is used where there was significant overdispersion as shown in Table A7.1.** | | | | | | |

# Appendix 8. Number of deaths in the alcohol cohort

## Table A8.1. Number of all-cause deaths for individuals with an alcohol-related hospital inpatient or emergency department presentation from 2005 to 2015 (N= 188,770).

|  | **Person-years** | | | **Observed deaths (n)** | | | **Expected** **deaths (n)** | | |
| --- | --- | --- | --- | --- | --- | --- | --- | --- | --- |
| **Age at death** | **Total** | **Males** | **Females** | **Total** | **Males** | **Females** | **Total** | **Males** | **Females** |
| All ages | 1,079,248 | 702,764 | 376,484 | 27,855 | 21,694 | 6,161 | 5,487 | 4,487 | 1,000 |
| 12-24 | 298,949 | 169,836 | 129,113 | 271 | 197 | 74 | 109 | 83 | 27 |
| 25-34 | 206,459 | 136,870 | 69,589 | 824 | 631 | 193 | 120 | 99 | 21 |
| 35-44 | 198,568 | 130,771 | 67,798 | 1,814 | 1,325 | 489 | 213 | 166 | 47 |
| 45-54 | 164,432 | 109,661 | 54,770 | 3,979 | 2,991 | 988 | 398 | 307 | 90 |
| 55-64 | 110,476 | 81,764 | 28,712 | 5,601 | 4,495 | 1,106 | 639 | 530 | 108 |
| 65-74 | 63,469 | 48,472 | 14,997 | 6,332 | 5,155 | 1,177 | 942 | 793 | 149 |
| 75-84 | 31,124 | 22,028 | 9,096 | 6,188 | 4,938 | 1,250 | 1,352 | 1,057 | 295 |
| 85+ | 5,770 | 3,362 | 2,409 | 2,846 | 1,962 | 884 | 714 | 451 | 262 |
| The number of expected deaths is computed by calculating the rate per person-years in the general population in each age-sex specific group (in the 12-15 years and 16-19 years age groups then in 5-year age groups from 15-19 years to 85-89 years, and 90+ years), then multiplying the rate by the observed person-years in the respective age-sex specific group. | | | | | | | | | |

## Table A8.2. Number of cause-specific deaths for the alcohol cohort from 2005 to 2013.

| **Cause of death** | **Observed deaths (n)** | | | **Expected** **deaths (n)** | | | **Percent of total (%)** | | |
| --- | --- | --- | --- | --- | --- | --- | --- | --- | --- |
|  | **Total**  (n=154,522) | **Males**  (n=102,620) | **Females**  (N=51,932) | **Total**  (n=154,522) | **Males**  (n=102,620) | **Females**  (N=51,932) | **Total**  (n=154,522) | **Males**  (n=102,620) | **Females**  (N=51,932) |
| **All deaths** | 20,529 | 16,035 | 4,494 | 3,009 | 2333 | 676 | 10.9 | 15.6 | 8.7 |
| **Attribution to alcohol†** |  |  |  |  |  |  |  |  |  |
| All causes *fully* attributable to alcohol | 5,205 | 4,113 | 984 | 102 | 92 | 10 | 3.4 | 4.0 | 2.1 |
| All causes *partly* attributable to alcohol | 15,838 | 12,428 | 3,410 | 2,150 | 1,652 | 497 | 10.2 | 12.1 | 6.6 |
| **Either fully or partly attributable to alcohol** | 17,082 | 13,349 | 3,733 | 2,177 | 1,676 | 501 | 11.1 | 13.0 | 7.2 |
| **1) Certain infectious and parasitic diseases (A00-B99)** | 2,918 | 2,313 | 605 | 263 | 207 | 56 | 1.9 | 2.3 | 1.2 |
| Tuberculosis | 19 | 17 | <10 | <10 | <10 | -- | <0.1 | <0.1 | -- |
| Viral hepatitis | 547 | 450 | 97 | 19 | 17 | <10 | 0.4 | 0.4 | 0.2 |
| **2) Neoplasms (C00-D48)** | 5,533 | 4,585 | 948 | 1,169 | 931 | 238 | 3.6 | 4.5 | 1.8 |
| Breast cancer | 146 | <10 | 140 | 47 | -- | 46 | 0.1 | 0.0 | 0.3 |
| Colon and rectal cancer | 406 | 346 | 60 | 112 | 89 | 22 | 0.3 | 0.3 | 0.1 |
| Oesophageal cancer | 208 | 170 | 38 | 37 | 33 | <10 | 0.1 | 0.2 | 0.1 |
| Cancer of lip and oral cavity, and pharynx | 187 | 160 | 27 | 14 | 12 | <10 | 0.1 | 0.2 | 0.1 |
| Liver cancer | 753 | 698 | 55 | 43 | 37 | <10 | 0.5 | 0.7 | 0.1 |
| **3) Blood and blood-forming organs (D50-D89)** | 621 | 459 | 162 | 83 | 63 | 20 | 0.4 | 0.4 | 0.3 |
| **4) Endocrine, nutritional and metabolic diseases (E00-E90)** | 2,854 | 2,320 | 534 | 446 | 346 | 101 | 1.8 | 2.3 | 1.0 |
| Diabetes mellitus | 1,799 | 1,529 | 270 | 306 | 244 | 62 | 1.2 | 1.5 | 0.5 |
| **5) Mental and behavioural disorders (F00-F99)** | 5,146 | 3,899 | 1,247 | 405 | 297 | 108 | 3.3 | 3.8 | 2.4 |
| Dementia | 1,424 | 1,022 | 402 | 234 | 155 | 79 | 0.9 | 1.0 | 0.8 |
| Alcohol use disorders | 2,729 | 2,175 | 554 | 57 | 52 | <10 | 1.8 | 2.1 | 1.1 |
| Other substance use disorders | 993 | 742 | 251 | 72 | 61 | 11 | 0.6 | 0.7 | 0.5 |
| **6) Disease of nervous system (G00-G99)** | 1,360 | 1,066 | 294 | 269 | 203 | 66 | 0.9 | 1.0 | 0.6 |
| Alzheimer's disease | 177 | 114 | 63 | 57 | 35 | 22 | 0.1 | 0.1 | 0.1 |
| **7) Diseases of the eye and adnexa (H00-H59)** | 41 | 26 | 15 | <10 | <10 | <10 | <0.1 | <0.1 | <0.1 |
| **9) Diseases of circulatory system (I00-I99)** | 9,876 | 7,891 | 1,985 | 1,638 | 1263 | 376 | 6.4 | 7.7 | 3.8 |
| Ischemic heart diseases | 3,887 | 3,206 | 681 | 765 | 622 | 143 | 2.5 | 3.1 | 1.3 |
| Stroke | 2,134 | 1,658 | 476 | 373 | 269 | 104 | 1.4 | 1.6 | 0.9 |
| Hypertensive heart disease | 128 | 103 | 25 | 23 | 18 | <10 | 0.1 | 0.1 | <0.1 |
| Cardiomyopathy & myocarditis | 618 | 544 | 74 | 52 | 45 | <10 | 0.4 | 0.5 | 0.1 |
| Atrial fibrillation & flutter | 1,063 | 842 | 221 | 177 | 130 | 47 | 0.7 | 0.8 | 0.4 |
| Heart failure | 1,829 | 1,431 | 398 | 321 | 236 | 85 | 1.2 | 1.4 | 0.8 |
| **10) Diseases of respiratory system (J00-J99)** | 6,478 | 5,018 | 1,460 | 885 | 694 | 191 | 4.2 | 4.9 | 2.8 |
| Chronic obstructive pulmonary disease | 2,800 | 2,131 | 669 | 290 | 238 | 52 | 1.8 | 2.1 | 1.3 |
| Asthma | 146 | 96 | 50 | 26 | 17 | <10 | 0.1 | 0.1 | 0.1 |
| **11) Diseases of digestive system (K00-K93)** | 6,365 | 5,042 | 1,323 | 304 | 240 | 64 | 4.1 | 4.9 | 2.5 |
| Cirrhosis & other chronic liver diseases | 5,273 | 4,195 | 1,078 | 135 | 115 | 20 | 3.4 | 4.1 | 2.1 |
| Esophagus, stomach and duodenum | 532 | 434 | 98 | 45 | 35 | 10 | 0.3 | 0.4 | 0.2 |
| Pancreatic diseases | 296 | 226 | 70 | 12 | <10 | <10 | 0.2 | 0.2 | 0.1 |
| **12) Skin and subcutaneous tissue (L00-L99)** | 293 | 218 | 75 | 37 | 26 | 10 | 0.2 | 0.2 | 0.1 |
| **13) Musculoskeletal system (M00-M99)** | 635 | 427 | 208 | 105 | 67 | 39 | 0.4 | 0.4 | 0.4 |
| **14) Diseases of genitourinary system (N00-N99)** | 2,349 | 1,858 | 491 | 372 | 287 | 85 | 1.5 | 1.8 | 0.9 |
| Acute kidney failure and chronic kidney disease | 2,040 | 1636 | 404 | 315 | 246 | 69 | 1.3 | 1.6 | 0.8 |
| **19)** **Injury and poisoning (S00-T98)** | 2,986 | 2,225 | 761 | 322 | 265 | 57 | 1.9 | 2.2 | 1.5 |
| Alcohol poisoning | 405 | 285 | 120 | 14 | 12 | 2 | 0.3 | 0.3 | 0.2 |
| **20) External causes of morbidity and mortality (V01-Y98)** | 3,180 | 2,380 | 800 | 360 | 295 | 65 | 2.1 | 2.3 | 1.5 |
| Suicide | 754 | 554 | 200 | 78 | 67 | 10 | 0.5 | 0.5 | 0.4 |
| Transport accidents | 201 | 172 | 29 | 49 | 42 | <10 | 0.1 | 0.2 | 0.1 |
| Interpersonal violence | 79 | 57 | 22 | <10 | <10 | <10 | 0.1 | 0.1 | <0.1 |
| The number of expected deaths is computed by calculating the rate per person-years in the general population in each age-sex specific group (in the 12-15 years and 16-19 years age groups then in 5-year age groups from 15-19 years to 85-89 years, and 90+ years), then multiplying the rate by the observed person-years in the respective age-sex specific group. | | | | | | | | | |

# Appendix 9. RECODE statement checklist

**The RECORD statement – checklist of items, extended from the STROBE statement, that should be reported in observational studies using routinely collected health data.**

|  | **Item No.** | **STROBE items** | **Location in manuscript where items are reported** | **RECORD items** | **Location in manuscript where items are reported** |
| --- | --- | --- | --- | --- | --- |
| **Title and abstract** | | | | | |
|  | 1 | (a) Indicate the study’s design with a commonly used term in the title or the abstract (b) Provide in the abstract an informative and balanced summary of what was done and what was found | 2 | RECORD 1.1: The type of data used should be specified in the title or abstract. When possible, the name of the databases used should be included.  RECORD 1.2: If applicable, the geographic region and timeframe within which the study took place should be reported in the title or abstract.  RECORD 1.3: If linkage between databases was conducted for the study, this should be clearly stated in the title or abstract. | 2 |
| **Introduction** | | | | | |
| Background rationale | 2 | Explain the scientific background and rationale for the investigation being reported | 4, 5 |  |  |
| Objectives | 3 | State specific objectives, including any prespecified hypotheses | 5 |  |  |
| **Methods** | | | | | |
| Study Design | 4 | Present key elements of study design early in the paper | 5 |  |  |
| Setting | 5 | Describe the setting, locations, and relevant dates, including periods of recruitment, exposure, follow-up, and data collection | 6 |  |  |
| Participants | 6 | *(a) Cohort study* - Give the eligibility criteria, and the sources and methods of selection of participants. Describe methods of follow-up | 6, 7 | RECORD 6.1: The methods of study population selection (such as codes or algorithms used to identify subjects) should be listed in detail. If this is not possible, an explanation should be provided.  RECORD 6.2: Any validation studies of the codes or algorithms used to select the population should be referenced. If validation was conducted for this study and not published elsewhere, detailed methods and results should be provided.  RECORD 6.3: If the study involved linkage of databases, consider use of a flow diagram or other graphical display to demonstrate the data linkage process, including the number of individuals with linked data at each stage. | 6, 7  10 |
| Variables | 7 | Clearly define all outcomes, exposures, predictors, potential confounders, and effect modifiers. Give diagnostic criteria, if applicable. | 7 – 9 | RECORD 7.1: A complete list of codes and algorithms used to classify exposures, outcomes, confounders, and effect modifiers should be provided. If these cannot be reported, an explanation should be provided. | 7 – 9 |
| Data sources/ measurement | 8 | For each variable of interest, give sources of data and details of methods of assessment (measurement).  Describe comparability of assessment methods if there is more than one group | 8, 9 |  |  |
| Bias | 9 | Describe any efforts to address potential sources of bias | N/A |  |  |
| Study size | 10 | Explain how the study size was arrived at | 10 |  |  |
| Quantitative variables | 11 | Explain how quantitative variables were handled in the analyses. If applicable, describe which groupings were chosen, and why | N/A |  |  |
| Statistical methods | 12 | (a) Describe all statistical methods, including those used to control for confounding  (b) Describe any methods used to examine subgroups and interactions  (c) Explain how missing data were addressed  (d) *Cohort study* - If applicable, explain how loss to follow-up was addressed  (e) Describe any sensitivity analyses | 9 |  |  |
| Data access and cleaning methods |  | .. | 9 | RECORD 12.1: Authors should describe the extent to which the investigators had access to the database population used to create the study population.  RECORD 12.2: Authors should provide information on the data cleaning methods used in the study. | 9  9 |
| Linkage |  | .. | 9 | RECORD 12.3: State whether the study included person-level, institutional-level, or other data linkage across two or more databases. The methods of linkage and methods of linkage quality evaluation should be provided. | 9 |
| **Results** | | | | | |
| Participants | 13 | (a) Report the numbers of individuals at each stage of the study (*e.g.*, numbers potentially eligible, examined for eligibility, confirmed eligible, included in the study, completing follow-up, and analysed)  (b) Give reasons for non-participation at each stage.  (c) Consider use of a flow diagram | (a) 10  (b) N/A  (c) 10 | RECORD 13.1: Describe in detail the selection of the persons included in the study (*i.e.,* study population selection) including filtering based on data quality, data availability and linkage. The selection of included persons can be described in the text and/or by means of the study flow diagram. | 10 |
| Descriptive data | 14 | (a) Give characteristics of study participants (*e.g.*, demographic, clinical, social) and information on exposures and potential confounders  (b) Indicate the number of participants with missing data for each variable of interest  (c) *Cohort study* - summarise follow-up time (*e.g.*, average and total amount) | (a) 10  (b) N/A  (c) 10 |  |  |
| Outcome data | 15 | *Cohort study* - Report numbers of outcome events or summary measures over time |  |  |  |
| Main results | 16 | (a) Give unadjusted estimates and, if applicable, confounder-adjusted estimates and their precision (e.g., 95% confidence interval). Make clear which confounders were adjusted for and why they were included  (b) Report category boundaries when continuous variables were categorized  (c) If relevant, consider translating estimates of relative risk into absolute risk for a meaningful time period | 10 – 12 |  |  |
| Other analyses | 17 | Report other analyses done—e.g., analyses of subgroups and interactions, and sensitivity analyses | N/A |  |  |
| **Discussion** | | | | | |
| Key results | 18 | Summarise key results with reference to study objectives | 11, 12 |  |  |
| Limitations | 19 | Discuss limitations of the study, taking into account sources of potential bias or imprecision. Discuss both direction and magnitude of any potential bias | 13, 14 | RECORD 19.1: Discuss the implications of using data that were not created or collected to answer the specific research question(s). Include discussion of misclassification bias, unmeasured confounding, missing data, and changing eligibility over time, as they pertain to the study being reported. | 12 – 16 |
| Interpretation | 20 | Give a cautious overall interpretation of results considering objectives, limitations, multiplicity of analyses, results from similar studies, and other relevant evidence | 13 – 16 |  |  |
| Generalisability | 21 | Discuss the generalisability (external validity) of the study results | 14 – 16 |  |  |
| **Other Information** | | | | | |
| Funding | 22 | Give the source of funding and the role of the funders for the present study and, if applicable, for the original study on which the present article is based | 17 |  |  |
| Accessibility of protocol, raw data, and programming code |  | .. | 5 | RECORD 22.1: Authors should provide information on how to access any supplemental information such as the study protocol, raw data, or programming code. | 5 |

*Reference: Benchimol EI, Smeeth L, Guttmann A, Harron K, Moher D, Petersen I, Sørensen HT, von Elm E, Langan SM, the RECORD Working Committee. The REporting of studies Conducted using Observational Routinely-collected health Data (RECORD) Statement. *PLoS Medicine* 2015; in press.

*Checklist is protected under Creative Commons Attribution ([CC BY](http://creativecommons.org/licenses/by/4.0/)) license.
